# Desiccation-Tolerant Vascular Plants: A Group of Species Largely Neglected in Conservation

**DOI:** 10.3390/plants14142184

**Published:** 2025-07-15

**Authors:** Wassila Ibrahim Seidou, Luiz Bondi, Stefan Porembski, Edson Lezin Bomisso

**Affiliations:** 1 UFR Biosciences, University Félix Houphouët-Boigny, Abidjan P.O. Box 582, Côte d’Ivoire; lebomisso@yahoo.fr; 2 West African Science Service Centre on Climate Change and Adapted Land Use (WASCAL), Graduate Research Programme on Climate Change and Biodiversity, Université Félix Houphouët-Boigny, Abidjan P.O. Box 582, Côte d’Ivoire; 3 Department of Botany, University of Rostock, 18059 Rostock, Germany; stefan.porembski@uni-rostock.de

**Keywords:** climate change, conservation, desiccation tolerance, diversity, land use change, protection areas, quarrying, resurrection plants, rock outcrops

## Abstract

The anthropogenic drivers of biodiversity loss are causing a global decline in biodiversity, yet some species remain overlooked in conservation efforts. In this study, we address the gaps between the current discussions and pressing issues on these species’ conservation. We focused on West Africa and used desiccation-tolerant vascular plants to conduct a systematic review of conservation discussions and a study case to assess their protection and exposure to quarrying and climate change. Our systematic review revealed, for the first time, that these plants are largely neglected in conservation discussions. Most species lack formal evaluations, and only four studies mentioned conservation, although without providing enough evidence to justify it. In our case study, we found biased protection among the species, with varying effectiveness of the protection areas against different anthropogenic threats. The two most exposed species were not present in protected areas, which were more effective at mitigating quarrying impacts than climate change. We highlight the need for conservation-explicit assessments and a mechanistic understanding of species’ conservation needs, such as quantitatively evaluating species vulnerability to threats, to support efficient conservation strategies. We call for conservation initiatives with specific objectives to include overlooked species in protected areas and adjust the conservation objectives to address specific threats.

## 1. Introduction

The world is experiencing high rates of biodiversity loss promoted by human activities [1,2,3]. However, drawing up global strategies for biodiversity conservation has been a challenging task because the anthropogenic drivers of biodiversity loss operate in different ways and species can be subjected to multiple drivers of strong intensities [1,4]. For example, land use change is mostly promoted by direct and local interventions on natural systems. On the other hand, climate change is indirectly triggered by global human activities, with some locations more exposed to changes regardless of their origin [5]. The intensity of both land use change and climate change is rising in some regions of the world such as West Africa [6,7], which includes areas recognized as global biodiversity hotspots [8]. The combination of multiple drivers increases the human pressures on species and their habitats and makes effective conservation strategies for biodiversity very much needed.

However, some fractions of biodiversity have been neglected in conservation. Desiccation-tolerant vascular plants (DT plants) are a good example of this. Also referred to as “resurrection plants” or “poikilohydrous”, DT plants have the remarkable ability to survive water loss up to 95% relative water content (equivalent to 0.1 g H_2_O/g dry weight) and resume their metabolism after rehydration with little or no biomass loss [9,10]. The desiccation tolerance ability is expected to provide an ecological advantage to thrive in environments with repetitive drought events throughout the year [9]. Due to this response, they can offer valuable genetic insights for breeding drought-resistant crops [11,12]. Also because of this response they are often seen as resistant species to environmental changes, and consequently neglected in conservation. However, this assumption is not justified by the current literature. Firstly, DT plants have been reported by earlier studies as sensitive to changes in the environmental conditions (e.g., changes in the length and intensity of drought might exceed the species’ capacity to tolerate desiccation) [9,13]. It means that anthropogenic drivers such as climate change can lead DT plants to not be able to tolerate desiccation in new climatic settings. Moreover, DT plants are associated with inselbergs, which are rock outcrops that abruptly rise from the landscape and act as island-like ecosystems to the species that inhabit them [9,10]. These ecosystems are often overlooked in conservation initiatives and are particularly exposed to land use change (e.g., quarrying) [14], and their insular characteristics [15] can reduce species’ capacity to migrate to new suitable areas in case of habitat loss. Due to these aspects, we believe that DT plants are a good model system to discuss the species neglected in conservation and the potential consequences of overlooking such conservation gaps.

In this study, we raise two research questions. (1) What is the state of knowledge in the conservation of DT plants? (2) Are the current protected areas addressing the conservation needs of DT plants? For this, we focus on West Africa and divide this study in two sections. In the first section, we conducted a systematic review to provide the first comprehensive list of DT plants for West Africa (including information about their distribution and conservation status) and to identify the discussions about DT plant conservation in the region. In the second section, we conducted a study case, in which we assess the protection and exposure of DT plants to two anthropogenic drivers of biodiversity loss: land use change and climate change. In this section, we compiled a list of DT plants reported in a database on West African inselbergs compiled by S. Porembski and compared their exposure to land use and climate change across unprotected and protected areas. We used quarrying activities and departures from the current climate conditions as proxies for land use and climate change, respectively. Despite being focused on DT plants from West Africa, we believe our study provides a basis for constructive discussion about the conservation of species often neglected in conservation and contributes valuable insights for generating more critical thinking about these species’ conservation.

## 2. Results

### 2.1. Diversity, Distribution, and Conservation of DT Plants in West Africa

All together, we found 49 desiccation-tolerant plant species in West Africa from 30 genera and 11 families across lycophytes, ferns, and angiosperms (Table 1). Asplenium stood out as the most species-rich genus, with six species reported, followed by Hymenophyllum and Sporobolus with four and three species, respectively. Poaceae emerged as the most species-rich, with ten species, followed by Pteridaceae and Hymenophyllaceae, respectively, with nine and eight species. Ferns represented 61.22%, angiosperms 36.73%, and lycophytes 2.04% of the species listed.

**Figure 1:**
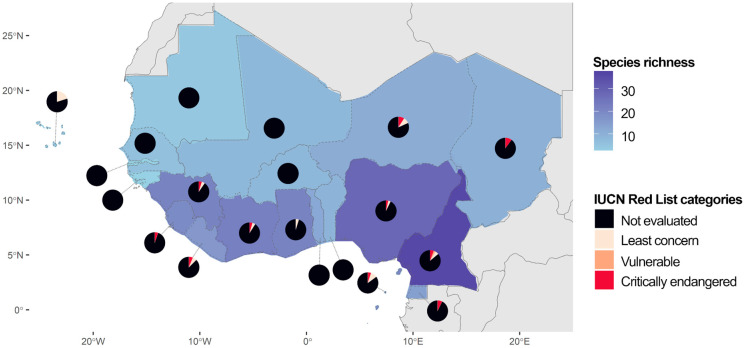
Number of desiccation-tolerant vascular plant species in West Africa per country (species richness) and their conservation status according to IUCN Red List categories.

Regarding the distribution of species in West Africa, Cameroon stood out as the country most rich in species (36), followed by Nigeria (28) and Côte d’Ivoire (22; Figure 1). The Gambia and Guinea-Bissau were the countries with the lowest number of species, having two species each. While some species were widespread in West Africa, others were restricted to one or a few countries. *Sporobolus festivus* (Poaceae) was the species most widespread in West Africa, being recorded in 15 countries. On the other hand, seven species were found in only one country in West Africa, including three nationally endemic species: *Afrotrilepis jaegeri* (Cyperaceae), endemic to Sierra Leone, and *Coleochloa domensis* (Cyperaceae) and *Craterostigma yaundense* (Linderniaceae), both endemic to Cameroon. In total, six species are endemic to West Africa. Besides *Afrotrilepis pilosa*, *A. jaegeri*, *C. domensis,* and *C. yaundense*, *Microdracoides squamosa* (Cyperaceae) and *Oropetium aristatum* (Poaceae) also have their distribution restricted to West Africa. Concerning the conservation status of the DT plants in West Africa, for the majority of the DT plants from West Africa, 42 species (86%) have not been evaluated for conservation by the IUCN. Only seven species (14%) have been evaluated, among which four species were classified as Least Concern (*Asplenium monanthes*, *Elaphoglossum acrostichoides*, *Cosentinia vellea*, and *Sporobolus stapfianus*; Aspleniaceae, Dryopteridaceae, Pteridaceae, and Poaceae, respectively), two species as vulnerable (*Asplenium aethiopicum* and *C. yaundense*; Aspleniaceae and Linderniaceae, respectively), and one species as critically endangered (*C. domensis*; Cyperaceae). A complete lack of assessment of DT plants for conservation was registered in eight countries in West Africa (40%). While 12 countries (60%) have at least one species assessed by IUCN, most species in those countries have not yet been assessed concerning their conservation.

**Figure 2:**
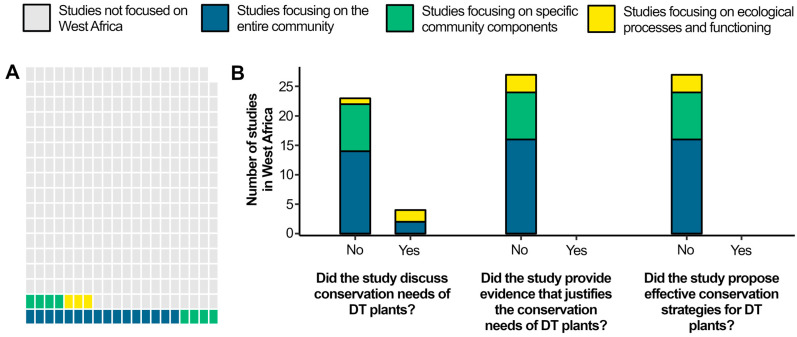
Number of studies on desiccation-tolerant vascular plants selected in our systematic review by 3 established categories. (**A**)—number of studies with explicit focus on West Africa; (**B**)—number of studies addressing conservation aspects of desiccation-tolerant plants in West Africa according to 3 questions raised.

Out of the 339 studies, only 27 studies (7.9%) were focused on West Africa (Figure 2; Table S4). Most of these 27 studies were focused on the entire community in which DT plants are embedded (16 studies, 4.7%). Eight studies focused on specific community components (2.3%), while only three studies focused on ecological processes and functioning (0.8%). Among those studies, only four (1.2% of studies, or 15% of studies on DT plants of West Africa) mentioned the conservation needs of DT plants [14,15,16,17], although no studies either produced enough evidence to justify the conservation needs of DT plants or proposed effective conservation strategies for DT plants. Of these four studies that mentioned the conservation needs about DT plants, two focused on the entire community [15,17], and two focused on the ecological processes and functioning [14,16] of the DT plants that have been found in an ecological context.

### 2.2. The Exposure of DT Plants to Anthropogenic Drivers of Biodiversity Loss and Their Protection on West African Inselbergs

**Figure 3:**
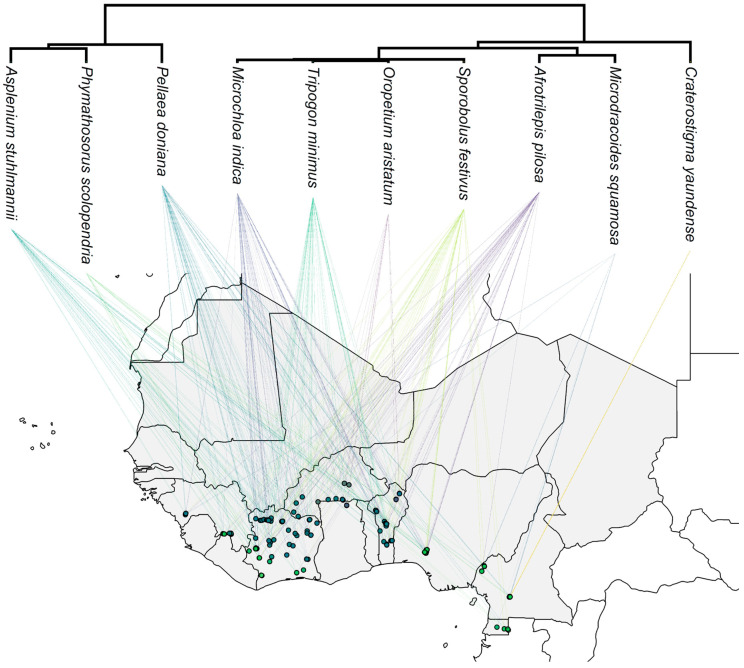
Uneven geographical distribution of 10 desiccation-tolerant vascular species across 123 inselbergs in West Africa.

We compiled a list of 123 inselbergs (spanning from Guinea to Equatorial Guinea) and selected 10 DT plants with an uneven and overlapping distribution across West African inselbergs: *Asplenium stuhlmannii* (Aspleniaceae); *Phymathosorus scolopendria* (Polypodiaceae); *Pellaea doniana* (Pteridaceae); *Afrotrilepis pilosa* and *Microdracoides squamosa* (Cyperaceae); *Microchloa indica*, *Oropetium aristatum*, *Tripogonella minimus*, and *Sporobolus festivus* (Poaceae); and *Craterostigma yaundense* (Linderniaceae; Figure 3, Table S5). These species form a diverse set of DT plants from taxonomic and geographical perspectives. For instance, while *A. stuhlmannii*, *P. doniana*, *S. festivus*, and *A. pilosa* have a broad-ranging distribution, *P. scolopendria*, *O. aristatum*, *M. squamosa*, and *C. yaundense* were registered on fewer inselbergs.

**Figure 4:**
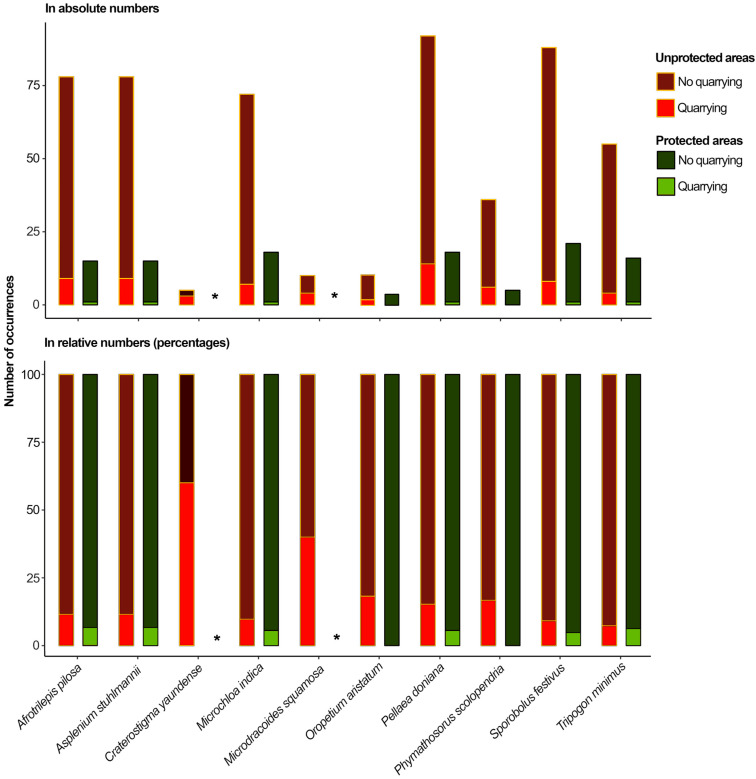
The impact of quarrying on the habitats of the 10 evaluated plant species. This figure illustrates the extent of exposure to quarrying for each species in unprotected and protected areas in both absolute and relative numbers. *—no presence of the species was registered in such areas.

While a significant difference between the unprotected and protected areas was registered in relation to the species’ exposure to quarrying, the species’ exposure to climate change was similar across the protected and unprotected inselbergs in all future scenarios (Table 2; a Mann–Whitney U test was conducted for species exposure to quarrying and *t*-test for species exposure to climate change). However, we could observe marked patterns among species protection and exposure to quarrying and climate change. Firstly, it is important to highlight that *C. yaundense* and *M. squamosa* were not present within the protected areas. Regarding the species’ exposure to quarrying, we found that all the species had at least one plant on an inselberg in which quarrying was registered (Figure 4; Table S6). While *T. minimus* had the lowest relative level of exposure to quarrying (7% of inselbergs, i.e., five out of seventy-one), *C. yaundense* showed the highest relative level of exposure to quarrying (60% of inselbergs, i.e., three out of five). The exposure of all the species to quarrying was reduced in absolute and relative values when inselbergs were included in the protected areas. While *T. minimus* had the lowest relative reduction in exposure to quarrying in protected areas (1.1 times less exposed to quarrying), *O. aristatum* and *P. scolopendria* had their exposure to quarrying reduced from 18% and 17%, respectively, to 0% (Table S7).

**Figure 5:**
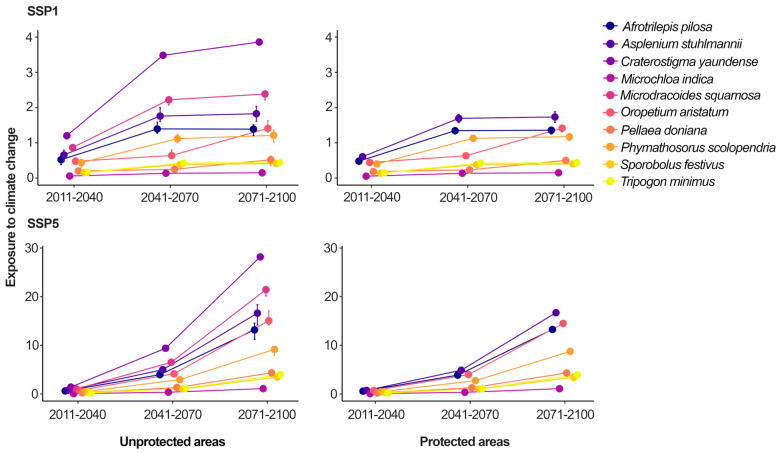
Projected climate change exposure of 10 desiccation-tolerant plants in West Africa under Low (SSP1) and High (SSP5) Emission Scenarios (2011–2100). This figure illustrates projected mean exposure of 10 desiccation-tolerant plant species to climate change under two different climate scenarios: SSP1, representing low-emission, sustainable pathway; and SSP5, high-emission, fossil-fuel-intensive pathway. Projections are shown across time-series: 2011–2040, 2041–2070, and 2071–2100.

Concerning the species’ exposure to climate change, all the species were more exposed to climate change in the most pessimistic scenario (SSP5) when compared to the most optimistic scenario (SSP1), especially in the farthest future (i.e., time-series 2071–2100; Figure 5, Table S8). *M. indica* was the species with least exposure to climate change, although its exposure scores increased 2.5-fold over time in the most optimistic scenario (i.e., increasing from 0.06 to 0.15 in the 2011–2040 and 2070–2100 time-series, respectively) and 18.3-fold in the most pessimistic scenario (i.e., increasing from 0.06 to 1.1 in the 2011–2040 and 2070–2100 time-series, respectively). On the other hand, *C. yaundense* was the species most exposed to climate change, reaching an exposure score 25.6 times higher than that of *M. indica* in the most pessimistic scenario of the farthest future. *C. yaundense* also showed greater rises in its exposure to climate change over time, increasing 3.2-fold in the most optimistic scenario (i.e., increasing from 1.2 to 3.86 in 2011–2040 and 2071–2100, respectively) and 19.7-fold in the most pessimistic scenario (i.e., increasing from 1.48 in 2011–2040 to 28.18 in 2071–2100).

## 3. Discussion

Our literature review reveals that DT plants form a diverse group in West Africa, but are a largely neglected group of species in conservation as are their habitats. Only seven species (14%) have been evaluated for conservation by the IUCN, and conservation issues with DT plants were only mentioned by four studies, although in an indirect way and without producing enough evidence to justify their need for conservation. We argue that conservation-explicit assessments are ultimately necessary to ensure the conservation of DT plants. Moreover, we recommend that instead of relying on general conservation narratives, future studies focus on a mechanistic understanding of species conservation needs in order to support efficient conservation strategies for DT plants.

Our case study revealed an important bias of protected areas towards certain species, besides its differential capacity to minimize the impact of the different anthropogenic drivers of biodiversity loss. Among the ten selected DT plants, two species (i.e., *M. squamosa* and *C. yaundense*) were not present within the protected areas. This is especially critical when considering that they are the species with the highest exposure rates to quarrying and climate change and are the two rarest species in our species pool. Conservation efforts must reduce such protection bias by explicitly including overlooked species in their conservation objectives. However, the conservation objectives must take into account the specific anthropogenic threats in each case. We found that the protected areas were more effective in mitigating the direct effects of human activities such as quarrying. However, they showed little effect on species exposure to climate change. We suggest, for example, using protected areas to ensure the connectivity among inselbergs as a more efficient strategy to cope with climate change.

### 3.1. The Need of Studies Specifically Designed to Assess the Conservation of DT Plants

Only four studies mentioned the need for the conservation of DT plants or their habitats. Two of them focused on the entire plant community (i.e., [15,17]), and two others focused on ecological processes and functioning (i.e., [14,16]). Even though they are not explicitly targeting DT plants, these studies either highlighted the ecological singularity of a species in the ecosystem DT plants occur (i.e., inselbergs), or acknowledged threats to its biodiversity in order to claim their need for conservation. However, those studies did not produce enough evidence to justify their claims. We acknowledge that it was not the aim of any of the mentioned studies to measure species conservation needs to support conservation efforts. However, we need studies explicitly designed to assess and discuss the conservation of DT plants. We believe conservation initiatives on DT plants would benefit from going beyond general conservation narratives towards a mechanistic understanding of species conservation needs.

The vulnerability framework suggested by Dawson et al. [18] is a good starting point to understand the mechanisms that lead DT plants to need conservation action, if they do. Dawson et al. [18] describe species vulnerability to environmental changes as a result of their exposure (i.e., the magnitude of changes a species undergoes), sensitivity (i.e., how much a species is affected by changes) and adaptive capacity (i.e., how much a species can mitigate the negative effects of environmental changes). According to this framework, species conservation needs increase under high-level exposure, high sensitivity, and low adaptive capacity. By identifying the importance of each component in the species vulnerability equation (i.e., exposure, sensitivity, and adaptive capacity), conservation initiatives can be more effective by strategically intervening in the critical component for the species in question. For example, we could assume that different DT plants in a same community are similarly exposed to a given threat. But the species’ sensitivity and adaptive capacity to this threat is likely to vary across species. Especially, we must consider that DT plants are found in 11 plant families in West Africa and desiccation tolerance has independently re-evolved across vascular plants’ phylogeny [19] (please also see Dollo’s Law [20]). If attempts to reduce species’ exposure to threats fail (e.g., local decision-makers cannot stop climate change effects in a given location), conservation strategies for species with high sensitivity, but high dispersal capacities (i.e., high adaptive capacity) might focus on ensuring species migration to new suitable locations (e.g., increasing the connectivity between protected areas and monitoring species movement and biotic interactions). However, if the species have high sensitivity and a low dispersal capacity (i.e., low adaptive capacity), more intensive conservation interventions might be taken to ensure the species will not go extinct in the wild (e.g., decision-makers could opt for assisted migration in order to guarantee that one species will not go extinct in the wild).

Understanding those three components is not always an easy task. Scientists and conservationists might encounter challenges in assessing some components (e.g., species can mitigate the negative effect of changes in many ways, from showing high genetic diversity to dispersing to new suitable areas) [18] and available resources are insufficient to evaluate every species at the proper time [21]. Studies with a focus on communities or their components could attempt to identify species to be prioritized for a posterior mechanistic conservation assessment. For DT plants, we have incomplete information about which species require more attention for conservation. We found that 86% of the species recorded have not yet been assessed for conservation under the IUCN Red List framework, as eight countries have not evaluated DT species; aligning with the general underrepresentation of West African plant species in global conservation assessments [22]. Although providing systematic and conservation-explicit guidelines for species conservation, the IUCN Red List framework might also be costly and time-consuming. For instance, monitoring population size reduction (Criteria A from the IUCN Red List framework) recommends measures over 10 years or three generations [23]. Alternatively, quicker (yet robust) assessments could also be used to inform about priority species for conservation (e.g., [24,25,26]) and which species the vulnerability framework from Dawson et al. [18] can be applied to for more effective conservation initiatives.

The studies we analyzed had a different approach. For example, some studies highlighted threats to inselberg species, including DT plants, to justify species conservation needs. Porembski [16] discussed that stress-tolerant native inselberg species can be outcompeted by invasive species with a better competitive ability. Invasive species will only establish themselves in a new community if they either have plant responses that confer greater fitness under local environmental constraints (i.e., “trying harder” strategy) or similar fitness when compared to that of local species (i.e., “joining the locals” strategy [27], but see also [28]). That means that in ecosystems in which environmental stress is supposed to be the strongest ecological constraint such as inselbergs, invasive species must either be fit enough to overcome environmental stress compared to local species or at least similarly fit in order to avoid being excluded by local species. Otherwise, we would be simply overestimating the impact of an alien species with neutral (or even positive) effects on the existing community [29,30]. We argue that invasive species with a better competitive ability would only be a threat in inselbergs if human pressures led to a shift in the importance of different ecological constraints. For example, over-visitation by humans or the introduction of grazing animals in inselbergs might intensify disturbance events (i.e., any external constraint that cause partial or total biomass loss; sensu Grime [31]) and reduce the relative importance of environmental stress (i.e., any external constraint that reduce plants productivity; sensu Grime [31]) in shaping inselberg plant communities. This shift would lead ruderal invasive species to exclude native species using the “trying harder” strategy because they are fitter under such new conditions (e.g., [32]). Thus, we support new studies focused on ecological processes that either monitor trends in species populations or habitat shifts over time with the presence of a potentially invasive species (e.g., [33]) or assess the changes in functional traits among native species due to the presence of newcomers (e.g., [34]). For DT plants, we believe that such studies could advance discussions about the invasiveness of inselbergs and their impact on DT plants.

Another common reasoning to justify the conservation needs of inselberg species, including DT plants, is that many plants exhibit particular responses to environmental challenges (e.g., “specializations” or “adaptations”) and that they have a restricted distribution (e.g., sometimes endemic to inselbergs). These two factors might reinforce the need for monitoring such species or ecosystems. However, there is not enough justification to prioritize a species for conservation. Concerning the particular responses to environmental challenges, we must keep in mind that such responses might also be beneficial to cope with some anthropogenic drivers of biodiversity loss. Desiccation tolerance allows for plants to overcome droughts with little or no biomass loss. If drought events become more frequent, intensive, and extensive owing to climate change, and thus lead plants to experience high dehydration or desiccation rates, one might logically argue that DT plants are not as sensitive to climate change as desiccation-sensitive plants. Regarding species-restricted distribution, the expectancy of higher extinctions risks for these species is often related to their narrow niche breadths unfolding a lower tolerance to changes and small population sizes, pushing species close to the viable population thresholds (i.e., minimum number of individuals required for the species to not go locally extinct) [35,36]. However, niche is not the only constraint to species distribution patterns (dispersal is also an important cause of the species restricted distribution). This means that species with restricted distribution are not necessarily sensitive to changes. Moreover, the species might be rare on a regional scale, but dominant on a local scale [37], so they are not close to the viable population thresholds. For instance, despite *A. pilosa* being endemic to West Africa and restricted to inselbergs, it can be present in very distinct environmental settings (e.g., locations where annual precipitation varies from less than 1000 to more than 3000 mm [38]) and achieve high abundances within local communities [39]. A species might indeed require conservation attention, but not necessarily because they have particular responses to the environment or because they have restricted distribution. We need more lines of evidence to evaluate species’ conservation needs rather than simply rely on clues that, despite their usefulness, may not appropriately describe species’ vulnerability to the anthropogenic drivers of biodiversity loss.

However, particular responses to environmental challenges (i.e., desiccation tolerance in our context) and restricted distribution are appealing features of DT plants and can be possibly used to encourage conservation awareness about DT plants. For example, the three Red Listed species *A. aethiopicum*, *C. domensis*, and *C. yaundense* could play an important role as Cinderella species in the context of DT plants in West Africa. *C. yaundense* is an inselberg-endemic species found only within the urban area of Yaoundé (the capital of Cameroon). Cinderella species are a type of surrogate species (i.e., species that are strategically used by conservation initiatives to promote the conservation of other species [40]) that are endangered, and despite not being as famous as flagship species, have enough charisma or public appeal to improve support on conservation initiatives [40,41,42]. These three species could be used in coordination to better capture the phylogenetic and ecological diversity specific to each area (e.g., as flagship fleets; see [43,44]). However, raising public awareness of plant species is trickier than for animals, but not an impossible task [45]. Alternative solutions should not be neglected, such as using umbrella species (i.e., another type of surrogate species, but which are more common features in the landscape, thus more likely to have stronger connections with local communities [46,47]), or other plant or animal species that occur on rock outcrops and would help conserving DT plants (since many DT plants are associated with these ecosystems). Moreover, we must keep in mind that the use of surrogate species in conservation initiatives risks us failing to solve complex problems through simplistic solutions [48]. Future studies are still necessary to select appropriate surrogate species and evaluate if such strategies satisfy local ecological realities.

### 3.2. Protected Areas Are Not Similarly Effective in Face of Different Conservation Challenges

**Figure 6:**
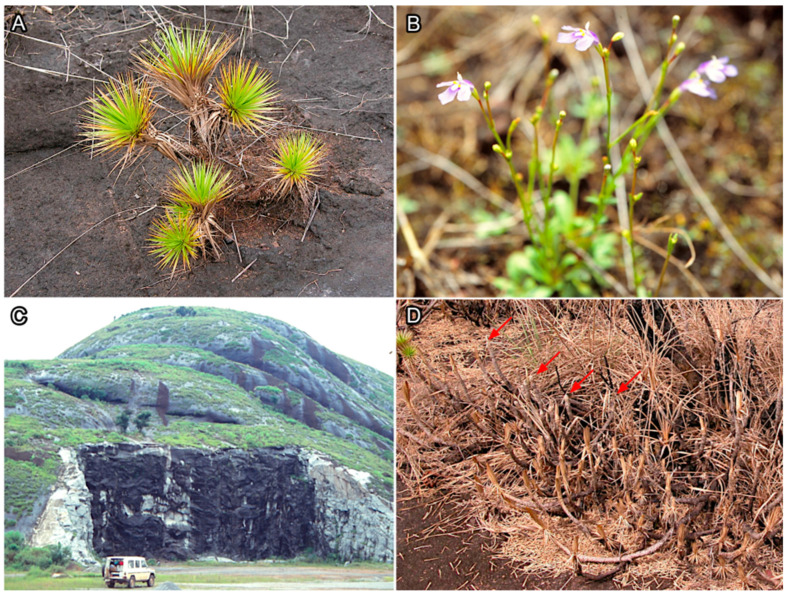
Species that most require conservation attention and suffer anthropogenic threats to their diversity are ones least favored by current protection areas. (**A**)—*Microdracoides squamosa* (Cyperaceae); (**B**)—*Craterostigma yaundense* (Linderniaceae); (**C**)—quarrying activities causing removal of desiccation-tolerant vascular plants; (**D**)—possible effects of climate change on desiccation tolerance ability of desiccation-desiccation vascular plants. Red arrows point to branches of *M. squamosa* that did not tolerate desiccation and rehydration cycles.

The species with more exposure to anthropogenic threats to biodiversity (i.e., *C. yaundense* and *M. squamosa*; Figure 6) are the ones not included in the existing protected area networks. This pattern is especially worrying if we consider that they are the two rarest species in our study, as this feature is linked with greater extinction risks [49,50]. Moreover, while *C. yaundense* is classified as vulnerable by the IUCN, *M. squamosa* is currently unevaluated by the IUCN, hindering our understanding about the conservation needs of one of the species that might most require our attention. Aichi Target 11 of the Convention on Biological Diversity [50] aimed to protect at least 17% of terrestrial areas by 2020 in a equable and ecologically representative way (and 30% by 2030 according to Target 3 of the Kunming-Montreal Global Biodiversity Framework) [51]. However, despite the 17% threshold reached in 2024 [52], our results show that ecological representativeness has not been yet met. It is estimated that nearly 60% of vascular plant diversity hotspots still remain outside protected areas, leaving many endemic and vulnerable species unprotected despite international conservation targets [53]. We believe that reducing the protection bias towards certain ecosystems and species requires the adjustment of conservation objectives to explicitly include overlooked species, such as *C. yaundense* and *M. squamosa*.

However, simply assigning protection areas might not be sufficient to ensure species protection against the anthropogenic drivers of biodiversity loss. In our study, the protected areas may have provided effective buffering against the direct effects of human activities such as quarrying (Table S7). However, they were less effective in mitigating the indirect effects of human activities such as climate change (Table S8). For instance, species like *O. aristatum* and *P. scolopendria* had their exposure to quarrying reduced to zero in the protected areas, but had roughly the same level of exposure to climate change when comparing the protected and unprotected inselbergs (in some futures scenarios, their level of exposure to climate change was higher in protected areas). Thus, the reasoning for using protected areas to reduce quarrying rates is more straightforward. By establishing protected areas, we can avoid quarrying activities in quarrying-prone areas, which are often neglected by conservation initiatives [14], and in which, species often have small geographic ranges [54]. Mitigating the impacts of quarrying may demand stricter land-use regulation and site-level protection. For example, some species were found on inselbergs included in a protected area, but with evidence of quarrying. Expanding the protection areas that include inselbergs and satisfy the conservation objectives (e.g., 30% under protection by 2030) requires enough resources to ensure the protection of established protected areas. We do not think it is the responsibility of researchers to find solutions for resource mobilization. But future studies on the conservation of DT plants could provide more evidence on the effectiveness of different conservation approaches to support resource mobilization campaigns, as recommended by the Analysis of the Strategic Plan 2011–2020 of the Convention on Biological Biodiversity [55].

Alternatively, protected areas should be planned more strategically when considering climate change. For example, we should not expect to stop climate change by demarking a protected area, although using protected areas to establish dispersal corridors for species’ possible migration is a more realistic strategy. There is a non-linear negative relationship between habitat loss and the persistence of species, which requires a habitat of minimal size and structure to maintain viable populations [56,57]. That is, a species’ probability of persistence gradually decreases with habitat loss until the connectivity among the remaining habitat patches in which a species exists reaches a point of no return for species persistence [57]. Here, habitat connectivity depends on factors, such as patch size and its spatial arrangement, besides how species interact with the landscape [57,58]. We believe that conservation planning that can cope with climate change can provide a better understanding of how habitat loss owing to climate change threatens species persistence. Future studies could evaluate the impact of inselberg size and isolation in species metapopulations as to assess the capacity of species to keep gene flow across different inselbergs. Ensuring connectivity among habitat patches where DT plants exist effectively is certain to combat other anthropogenic threats, but we argue it should be more explicitly established as one of the protection area objectives against climate change.

Our study considered only exposure to anthropogenic threats, offering a partial picture of species vulnerability. The fact that the two most exposed species are the most rare and unprotected ones should raise our awareness, but it is noteworthy to mention that high-level exposure does not necessarily translate into higher extinction risks. For example, although highly exposed to climate change, *O. aristatum*, an annual species, might be able to complete its life cycle before extreme climatic conditions and escape, to some extent, the negative effects of climate change. On the other hand, species might face high extinction risks even under modest exposure due to their narrower ecological tolerances [59]. For instance, most Hymenophyllaceae species occur as epiphytes in moist forests and are often sensitive to changes in the desiccation rate due to the lack mechanisms to reduce water loss (e.g., lack of cuticles) [60]. Alternatively, different anthropogenic threats can act in combination, causing additive effects on each other and intensifying their impact on species [3]. Moreover, we only used two anthropogenic threats as proxies for the different drivers of biodiversity loss. For instance, *M. indica* showed the least exposure to climate change, but its broader occurrence across different ecosystems might expose this species to threats that inselberg-endemic species are not subjected. Lastly, we also advocate for future studies that not only use multiple lines of evidence to assess species vulnerability, but that also encompass a greater array of threats (e.g., direct exploitation and invasive alien species) to improve our understanding of species conservation needs and support efficient conservation initiatives.

## 4. Materials and Methods

### 4.1. Study Area

For the purposes of this study, we considered West Africa as a biogeographic region extending from Senegal to Equatorial Guinea, following a broad ecological definition that includes approximately 20 countries and encompasses humid and transitional vegetation zones, as described by the *Encyclopædia Britannica* [61]. This includes countries such as Cameroon, Chad, and Equatorial Guinea, which are politically classified as part of Central Africa, but share strong ecological and floristic continuity with the West African forest zone. This area spans a strong climatic gradient, with annual rainfall ranging from 250 mm in the arid north to 3000 mm in the humid forest regions. As a result, there are four major vegetation zones in West Africa: the Guineo-Congolian Zone (regional center of endemism), the Guineo-Congolian/Sudanian Zone (transitional zone), the Sudanian Zone (regional center of endemism), and the Sahel Zone (transitional zone). One of the most important ecosystems in this region is the Guinean Coastal forest, which served as a refuge for many species during historical climate fluctuations in the Pleistocene. Today, it remains one of Africa’s eight biodiversity hotspots, characterized by high species endemicity. However, only 15% of its original forest cover remains [62], motivating conservation efforts, in which 90% of national protected areas are forests [63]. Inselbergs are usually overlooked, unless embedded in protected areas.

### 4.2. Desiccation-Tolerant Vascular Plants in West Africa

First, we conducted a systematic literature review in order to obtain a list of DT vascular plants and to evaluate their conservation concerns in West Africa. For this, we applied the key-word combination “desiccation tolerant” OR “resurrection” AND angiosperm* OR pteridophyte* OR lycophyte* OR vascular OR plant*” for the search engines (1) Scopus, (2) Web of Science, (3) Academia, and (4) Google Scholar from 1950 to 2024. We initially retrieved a total of 1657 documents (243 documents from Scopus, 567 from Web of Science, 36 from Academia, and 811 from Google Scholar). After compiling this literature pool, we started cleaning it by removing 549 duplicated records. Then, we removed 1322 documents that were either not subjected to a peer-reviewed process (e.g., grey literature), or which were not considered in the scope of our study, such as publications that did not focus on plants as their main research objective (e.g., studies on geology or zoology). After this step, an addition of 4 articles were included in our literature pool [16,64,65,66]. They were considered important articles in the context of our study and were not found by the abovementioned key-word search. In total, 339 publications were retained and used in our analyses.

We compiled a comprehensive list of desiccation-tolerant plant species in West Africa. Additionally, we provided information about their geographical distribution in West Africa and conservation status. Information on the distribution of these species in West Africa was compiled from *The Flora of West Africa* [67], *Flora of savannas and rock outcrops of Côte d’Ivoire* [68], and Plants of the World Online [69]. Here, we used their occurrence at the country level to discuss species distribution patterns and estimate the diversity patterns of DT plants in West Africa (calculating species richness by country, i.e., number of different species occurring in the same country). The following countries were considered in our study: Benin, Burkina Faso, Côte d’Ivoire, Cameroon, Cabo Verde, Chad, the Gambia, Ghana, Guinea, Guinea-Bissau, Equatorial Guinea, Liberia, Mali, Mauritania, Niger, Nigeria, Senegal, Sierra Leone, and Togo. In this study, we followed the classification of Plants of the World Online [69] and grouped into the Gulf of Guinea, the islands of Bioko, Príncipe, São Tomé, and Annobón.

About their conservation status, we examined species inclusion in the IUCN Red List of Threatened Species version 2025-1 [70]. We used the IUCN Red List 9 categories. The categories (i) extinct and (ii) extinct in the wild indicate species are either found extinct, or only have individuals living outside their natural habitat. Species threatened with extinction are found in the categories (iii) critically endangered, (iv) endangered, and (v) vulnerable, in which their extinction risk decreases in the order given. Those categorized as (vi) near-threatened and (vii) Least Concern are not considered as being at risk of extinction. At last, a lack of assessment exists for species in the categories (viii) data-deficient and (ix) not evaluated, which have insufficient information for assessment, or simply have not yet been assessed for conservation.

Then, to evaluate the current state of research and discussion about the conservation of DT plants in West Africa, we calculated the number of studies that focused on West Africa and raised three questions: (i) Did the study discuss the conservation needs of DT plants? (ii) Did the study produce enough evidence to justify the conservation needs of DT plants? (iii) Did the study propose effective conservation strategies for DT plants? For instance, we considered that the study discussed conservation needs for DT plants if it identified DT plants as vulnerable or as strategic components for biological conservation. Using this example, we considered that the study produced enough evidence to justify the conservation needs of DT plants if it quantitatively measured the vulnerability of species or its relevance to any facet of diversity in the ecological contexts they occur (i.e., either taxonomic, phylogenetic, or functional diversity). For the third question, we considered that this study proposed effective conservation strategies for DT plants if it suggested, for example, priority species or locations for conservation. To improve our understanding about patterns among those studies, we grouped them in three categories: (1) studies focusing on the entire community, (2) studies focusing on specific community components, and (3) studies focusing on ecological processes and functioning. While we included in the first group studies that focus on the diversity and ecological patterns of the whole community, in the second group, we included studies in which such patterns are discussed from the perspective of particular habitat types or taxonomic/functional groups. Lastly, we included in the third group of studies whose main objective was to discuss the ecological phenomena that explain how community patterns come about, either focusing on the whole community or specific community components.

### 4.3. Study Case to Evaluated the Exposure of DT Plants to Land Use Change and Climate Change

We compiled a list of DT plants reported by S. Porembski in a database on West African inselbergs as a result of extensive fieldwork in the region from 1986 to 2024. Only the inselbergs from this database were included in all our analyses. First, as a rough descriptor of the conservation efforts for each species, we counted the number of inselbergs in which each species exist that are included in protected areas and other effective area-based conservation measures using the World Database on Protected Areas [52]. Here, inselbergs in which species exist were categorized into two groups, protected and unprotected.

We focused on species exposure to two anthropogenic drivers of biodiversity loss: land use change and climate change. First, we use optical evidence of quarrying activities on the inselbergs the selected DT plants exist on to estimate species exposure to land use change. For this, we used Google Earth Engine, a cloud-based geospatial analysis platform [71]. Sentinel-2 imagery (QA60 band) was retrieved, and a 2 km radius buffer was applied around each focal location and the surrounding area based on the geographical coordinates of inselbergs from the database of S. Porembski. Then, we produced a composite image for the months between October and December of the two consecutive years of 2022 and 2023, applying a cloud mask (cloud cover of less than 5%) to ensure data quality. The selection of the same months across consecutive years (2022 and 2023) minimizes seasonal variability, allowing us to better compose the obtained images. The period from October to December was chosen because cloud cover was reduced, which allowed us to use more images with a cloud cover < 5%, improving image clarity and the quality of our composite images.

Then, we used the departures in climatic variables for the inselbergs the selected DT plants exist on to estimate their exposure to climate change. For this, we conducted climate niche factorial analysis [72] to obtain information about shifts in climatic variables within the inselbergs. We first modeled species distribution for each species using the distribution records of species available in the database Global Biodiversity Information Facility [73] (see Table S1). The species occurrence data were harmonized, eliminating duplicates, errors, and uncertain geographic entries. We used the information provided by the database Plants of the World Online [69] as a reference for the natural occurrence of species. We also retained records that despite a lack of geographic coordinates, included detailed location information. Here, we employed the centroid of the municipality. To model species distribution, we used the modelling technique MaxEnt [74] and the bioclimatic variables available in the CHELSA dataset (Table S2) [75] at a spatial resolution of 2°30′. We excluded bioclimatic variables 6, 9, 10, and 11, representing temperature extremes and seasonal means, due to their high collinearity with the other variables. Removing them reduces redundancy and helps avoid overfitting in climate-based models, ensuring more robust and interpretable results. The MaxEnt technique was chosen because of its high predictability to identify suitable areas of occurrence for species from a niche-based perspective, even for taxa with only a few occurrence data [76]. We used only one observation within a 1 km radius and selected the most important environmental variables for each modelled species with a low level of correlation. These steps were considered in order to mitigate sampling bias effects (assuming that species populations are found within isolated rock outcrops and more than one sampling point can be registered for the same inselberg) and to avoid issues with multicollinearity and model overfitting. As for final distribution, we used the consensus of binary maps (i.e., presence–absence) between at least 50% of 5 ensemble MaxEnt models, in which accuracy was higher than 0.8 and 0.6 according to the receiver operator characteristic curve and true skill statistic tests, respectively. To produce the binary maps, we used the individual model thresholds given by MaxEnt in order to maximize the true positive and true negative rates. The evaluation of model accuracy was performed with cross-validation using the k-means method (k = 5), in which we used 100, 1000, or 10,000 random background points if the species had less than 30, less than 300, or at least 300 occurrence points, respectively [77].

We conducted climate niche factorial analysis using the same most important bioclimatic variables that explain each species’ distribution. In this analysis, the present conditions were compared to the future conditions. For the future, two Representative Concentration Pathway scenarios were used, with SSP1 being the less-severe (sustainable path with environmental boundaries and low greenhouse gas emission) and SSP5 being the worst (very high greenhouse gas emissions, with fossil-fueled development). Also, three intervals of time-series were considered: 2011–2040, 2041–2070, and 2071–2100. Climate niche factorial analysis was chosen for its ability to calculate departures from the prevailing climatic conditions across grid cells and provide a single exposure value for each species to climate change. This overall value of departure allows for robust comparison between species, especially when different variables were selected in order to take into consideration species individual sensitivities to environmental conditions [72]. Lastly, we extracted the exposure values for the inselbergs each species exists on.

To compare DT plants’ exposure to quarrying and climate change across unprotected and protected areas, we performed a *t*-test when our data was normally distributed and homoscedastic, or a Mann–Whitney U test when our data did not satisfy the assumptions of parametric statistics. Since hypotheses tests were repeated to include different climate change scenarios, we adjusted the *p*-values using Bonferroni correction. For graphic representation purposes, we produced a phylogenetic tree using the phylogenetic hypothesis provided by Jin and Qian (as *Scenario 3*) [78]. All analyses were conducted and all graphical representations were created using the software R, version R.4.2.2 (please see Table S3 for the used R packages) [79].

## 5. Conclusions

This study provides the first comprehensive list of DT plants in West Africa and reveals, for the first time, a significant research gap related to the conservation of these species. Only 14% of the species have been evaluated for conservation by the IUCN, and only 15% of studies on DT plants of West Africa have discussed their conservation needs, but without producing enough evidence to justify their need for conservation. This is especially critical when considering that among the species we assessed, the two species with the lowest level of protection are the ones with the most exposure to quarrying and climate change. Our study highlights an important bias of protected areas towards certain species, and their differential capacity to minimize the impact of the different anthropogenic drivers of biodiversity loss. Protection areas might be effective to reduce quarrying activities in inselbergs, but they likely will not avoid the negative effects of climate change on species. We believe that a similar situation might be found for other species neglected in conservation like DT plants, and our suggestions to make conservation initiatives more effective can also apply to other groups of species. For example, we advocate for more conservation-explicit assessments and a mechanistic understanding of species’ conservation needs. This can be conducted by quantitatively evaluating the different components of species’ vulnerability to threats (i.e., species exposure, sensitivity, and adaptive capacity). By doing this, we scientists and conservationists could better inform decision-makers about adequate conservation strategies. Alternative conservation strategies, such the use of surrogate species for conservation, should not be ruled out. But, more importantly, future conservation initiatives must be drawn up with a clearer and more specific objective to include overlooked species, adjust specific actions to address specific threats, and satisfy species conservation needs.

## Figures and Tables

**Table 1:** Checklist of desiccation-tolerant vascular plants present in West Africa, their distribution in West Africa, and conservation status according to IUCN Red List. BEN—Benin; BFA—Burkina Faso; CIV—Côte d’Ivoire; CMR—Cameroon; CPV—Cabo Verde; GGN—Gulf of Guinea Is.; GHA—Ghana; GIN—Guinea; GMB—the Gambia; GNB—Guinea-Bissau; GNQ—Equatorial Guinea; LBR—Liberia; MLI—Mali; MRT—Mauritania; NER—Niger; NGA—Nigeria; SEN—Senegal; SLE—Sierra Leone; TCD—Chad; TGO—Togo.

			Distribution in West Africa	IUCN Red List Categories
Lycophytes				
	Selaginellaceae			
		*Selaginella njamnjamensis* Hieron.	BEN, CMR, MLI, NGA	Not evaluated
Pteridophytes				
	Aspleniaceae			
		*Asplenium aethiopicum* (Burm.f.) Becherer	CIV, CMR, GGN, GIN, GNQ, LBR, NER, NGA, SLE, TCD	Vulnerable
		*Asplenium friesiorum* C.Chr.	CMR, GGN, NGA	Not evaluated
		*Asplenium megalura* Hieron.	CIV, CMR, GHA, GGN, GIN, LBR, SLE, TGO	Not evaluated
		*Asplenium monanthes* L.	CMR, GGN	Least Concern
		*Asplenium sandersonii* Hook.	CMR, GGN, GNQ, NGA	Not evaluated
		*Asplenium stuhlmannii* Hieron.	CIV, CMR, GIN, NGA, SLE	Not evaluated
	Dryopteridaceae			
		*Elaphoglossum acrostichoides* (Hook. & Grev.) Schelpe	CIV, CMR, GGN, GHA, GIN, LBR	Least Concern
	Hymenophyllaceae			
		*Crepidomanes chevalieri* (Christ) Ebihara & Dubuisson	CIV, CMR, GGN, GHA, GIN, LBR, NGA, SLE	Not evaluated
		*Crepidomanes melanotrichum* (Schltdl.) J.P.Roux	CIV, CMR, GGN, GHA, GIN, LBR, NGA, SLE	Not evaluated
		*Didymoglossum erosum* (Willd.) Beentje	CIV, CMR, GHA, GIN, LBR, NGA, SLE	Not evaluated
		*Hymenophyllum capillare* Desv.	CMR, GGN, GHA	Not evaluated
		*Hymenophyllum hirsutum* (L.) Sw.	CMR, GGN, GHA, GIN, GNQ, CIV, LBR	Not evaluated
		*Hymenophyllum kuhnii* C.Chr.	CMR, GGN, GHA, GIN, GNQ, LBR, NGA, SLE	Not evaluated
		*Hymenophyllum splendidum* Bosch	CMR, GNQ	Not evaluated
		*Polyphlebium borbonicum* (Bosch) Ebihara & Dubuisson	CIV, CMR, GGN, GNQ, GHA, GIN, LBR	Not evaluated
	Polypodiaceae			
		*Loxogramme abyssinica* (Baker) M.G.Price	CIV, CMR, GGN, GHA, GIN, GNQ, LBR, NGA, SLE, TGO	Not evaluated
		*Melpomene flabelliformis* (Poir.) A.R.Sm. & R.C.Moran	CMR, GGN	Not evaluated
		*Phymatosorus scolopendria* (Burm.f.) Pic.Serm.	BEN, CIV, CMR, GGN, GNQ, GHA, GIN, LBR, NGA, SLE, TGO	Not evaluated
		*Platycerium stemaria* (P.Beauv.) Desv.	BEN, CIV, CMR, GGN, GNQ, GHA, GIN, LBR, NGA, SEN, SLE	Not evaluated
		*Pleopeltis macrocarpa* (Willd.) Kaulf.	CIV, CMR, GGN, GIN, LBR, NGA, SLE	Not evaluated
	Pteridaceae			
		*Actiniopteris radiata* (Sw.) Link	CMR, CPV, MLI, NGA, TCD, TGO	Not evaluated
		*Actiniopteris semiflabellata* Pic.Serm.	MRT	Not evaluated
		*Adiantum incisum* Forssk.	CIV, CMR, CPV, GHA, NGA, TGO	Not evaluated
		*Cheilanthes coriacea* Decne.	NER, TCD	Not evaluated
		*Cheilanthes inaequalis* Mett.	CMR, GIN, NGA	Not evaluated
		*Cosentinia vellea* (Aiton) Tod.	CPV	Least Concern
		*Hemionitis farinosa* (Forssk.) Christenh.	CMR, GGN, NGA, SLE	Not evaluated
		*Pellaea doniana* Hook.	GNQ	Not evaluated
		*Vittaria guineensis* Desv.	CIV, CMR, GGN, GHA, GIN, GNQ, LBR, NGA, SLE, TGO	Not evaluated
	Tectariaceae			
		*Arthropteris orientalis* (J.F.Gmel.) Posth.	CIV, CMR, GGN, GHA, GIN, LBR, NGA, SLE	Not evaluated
Angiosperms				
	Cyperaceae			
		*Afrotrilepis jaegeri* J.Raynal	SLE *	Not evaluated
		*Afrotrilepis pilosa* (Boeckeler) J.Raynal	BEN, BFA, CIV, CMR, GHA, GIN, GNQ, LBR, MLI, NGA, SEN, SLE, TGO	Not evaluated
		*Coleochloa abyssinica* (Hochst. ex A.Rich.) Gilly	CMR, NGA	Not evaluated
		*Coleochloa domensis* Muasya & D.A.Simpson	CMR *	Critically endangered
		*Microdracoides squamosa* Hua	CMR, GIN, NGA, SLE *	Not evaluated
	Linderniaceae			
		*Craterostigma plantagineum* Hochst.	BFA, NER, TCD	Not evaluated
		*Craterostigma yaundense* (S.Moore) Eb.Fisch., Schäferh. & Kai Müll.	CMR *	Vulnerable
	Poaceae			
		*Microchloa indica* (L.f.) P.Beauv.	BEN, BFA, CIV, CMR, GHA, GIN, GNB, MLI, NER, NGA, SEN, SLE, TCD, TGO	Not evaluated
		*Microchloa kunthii* Desv.	BEN, BFA, CIV, CMR, GHA, NGA, TGO	Not evaluated
		*Oropetium aristatum* (Stapf) Pilg.	BEN, BFA, CIV, GHA, GMB, GNB, MLI, NER, SEN, TGO *	Not evaluated
		*Oropetium capense* Stapf	MLI, MRT, NER, TCD	Not evaluated
		*Sporobolus festivus* Hochst. ex A.Rich.	BEN, BFA, CIV, CMR, GHA, GIN, GMB, GNQ, MLI, MRT, NER, NGA, SEN, TCD, TGO	Not evaluated
		*Sporobolus pellucidus* Hochst.	BFA, NER, TCD	Not evaluated
		*Sporobolus stapfianus* Gand.	NER, NGA	Least Concern
		*Tripogon major* Hook.f.	SLE	Not evaluated
		*Tripogon multiflorus* Miré & H.Gillet	CPV, NER, TCD	Not evaluated
		*Tripogonella minima* (A.Rich.) P.M.Peterson & Romasch.	BEN, BFA, CIV, CMR, CPV, GHA, MLI, MRT, NER, NGA, SEN, TCD, TGO	Not evaluated
	Velloziaceae			
		*Xerophyta schnitzleinia* (Hochst.) Baker	NGA, GNQ	Not evaluated

* endemic to West Africa.

**Table 2:** Comparison between unprotected and protected areas in relation to species exposure to quarrying and climate change. We conducted Mann–Whitney U test to analyze species’ exposure to quarrying and *t*-test to analyze species’ exposure to climate change. *p*-values for species exposure to climate change were adjusted by Bonferroni correction.

			W-Value/t-Value	*p*-Value
Quarrying			80	0.0004
Climate change				
	SSP1			
		2011–2040	1.17	0.7809
		2041–2070	1.04	0.9456
		2071–2100	1.05	0.9297
	SSP5			
		2011–2040	1.1	0.8592
		2041–2070	1.03	0.9519
		2071–2100	0.93	1

## Data Availability

The original contributions presented in this study are included in this Article/Supplementary Material. Further inquiries can be directed to the corresponding author(s).

## Supplementary Materials

The following supporting information can be downloaded at: https://www.mdpi.com/article/10.3390/plants14142184/s1. Table S1. Reference of occurrence datasets retrieved from GBIF database. Table S2: Bioclimatic variables retrieved from CHELSA database and used in this study. Table S3: List of R packages used in this study. Table S4: Studies about desiccation-tolerant vascular plants in West Africa. Q1—Did the study discuss conservation needs of DT plants?; Q2—Did the study provide enough evidence that justifies the conservation needs of DT plants?; Q3—Did the study propose effective conservation strategies for DT plants?; Categories: I—Studies focusing on the entire community; II—studies focusing on specific community components; III—studies focusing on ecological processes and functioning. Table S5: Species presence (1) and absence (0) across 123 inselbergs in West Africa. Ap—*Afrotrilepis pilosa*, As—*Asplenium stuhlmannii*, Cy—*Craterostigma yaundense*, Mi—*Microchloa indica*, Ms—*Microdracoides squamosa*, Oa—*Oropetium aristatum*, Pd—*Pellaea doniana*, Ps—*Phymathosorus scolopendria*, Sf—*Sporobolus festivus*, Tm—*Tripogonella minimus.* Table S6: Species exposure to two anthropogenic drivers of biodiversity loss: quarrying and climate change. The evaluated species are *Asplenium stuhlmannii* (Aspleniaceae), *Phymathosorus scolopendria* (Polypodiaceae), *Pellaea doniana* (Pteridaceae), *Afrotrilepis pilosa* and *Microdracoides squamosa* (Cyperaceae), *Microchloa indica*, *Oropetium aristatum*, *Tripogonella minimus*, and *Sporobolus festivus* (Poaceae), and *Craterostigma yaundense* (Linderniaceae). Table S7: Species exposure to quarrying when comparing unprotected and protected inselbergs. The evaluated species are *Asplenium stuhlmannii* (Aspleniaceae), *Phymathosorus scolopendria* (Polypodiaceae), *Pellaea doniana* (Pteridaceae), *Afrotrilepis pilosa* and *Microdracoides squamosa* (Cyperaceae), *Microchloa indica*, *Oropetium aristatum*, *Tripogonella minimus*, and *Sporobolus festivus* (Poaceae), and *Craterostigma yaundense* (Linderniaceae). Table S8: Species exposure to climate change when comparing unprotected and protected inselbergs. The evaluated species are *Asplenium stuhlmannii* (Aspleniaceae), *Phymathosorus scolopendria* (Polypodiaceae), *Pellaea doniana* (Pteridaceae), *Afrotrilepis pilosa* and *Microdracoides squamosa* (Cyperaceae), *Microchloa indica*, *Oropetium aristatum*, *Tripogonella minimus*, and *Sporobolus festivus* (Poaceae), and *Craterostigma yaundense* (Linderniaceae).

## Abbreviations

The following abbreviations are used in this manuscript:

DT	Desiccation-tolerant
IUCN	International Union for Conservation of Nature
SSP	shared socioeconomic pathway
