# Supplementary material for: Desiccation-Tolerant Vascular Plants: A Group of Species Largely Neglected in Conservation
Source: Plants (Basel). 2025 Jul 15;14(14):2184. doi: 10.3390/plants14142184 (PMC12298277; doi:10.3390/plants14142184)
Supplement: Supplementary file 1 [file plants-14-02184-s001.zip › plants-3716266-supplementary.pdf]

plants

MDPI

*Supplementary material*

# Desiccation-Tolerant Vascular Plants: a Group of Species Largely Neglected for Conservation

**Wassila Ibrahim Seidou, Luiz Bondi, Stefan Porembski and Edson Lezin Bomisso**

**Table S1.** Reference of occurrence datasets retrieved from GBIF database.

| Species | GBIF (references) |
|---|---|
| *Actiniopteris radiata* | GBIF.org (22 March 2023) GBIF Occurrence Download https://doi.org/10.15468/dl.w7xj3c |
| *Actiniopteris semiflabellata* | GBIF.org (22 March 2023) GBIF Occurrence Download https://doi.org/10.15468/dl.u46p8v |
| *Adiantum incisum* | GBIF.org (22 March 2023) GBIF Occurrence Download https://doi.org/10.15468/dl.m4pmmc |
| *Afrotrilepis jaegeri* | GBIF.org (28 May 2025) GBIF Occurrence Download https://doi.org/10.15468/dl.7nukjh |
| *Afrotrilepis pilosa* | GBIF.org (18 February 2024) GBIF Occurrence Download https://doi.org/10.15468/dl.vmqcx8 |
| *Allosorus coriacea* | GBIF.org (22 March 2023) GBIF Occurrence Download https://doi.org/10.15468/dl.5sremd |
| *Arthropteris orientalis* | GBIF.org (22 March 2023) GBIF Occurrence Download https://doi.org/10.15468/dl.yfu9nx |
| *Asplenium aethiopicum* | GBIF.org (22 March 2023) GBIF Occurrence Download https://doi.org/10.15468/dl.hjqzcy |
| *Asplenium friesiorum* | GBIF.org (22 March 2023) GBIF Occurrence Download https://doi.org/10.15468/dl.gen6n3 |
| *Asplenium megalura* | GBIF.org (22 March 2023) GBIF Occurrence Download https://doi.org/10.15468/dl.uweaw5 |
| *Asplenium monanthes* | GBIF.org (22 March 2023) GBIF Occurrence Download https://doi.org/10.15468/dl.6c9ztx |
| *Asplenium sandersonii* | GBIF.org (22 March 2023) GBIF Occurrence Download https://doi.org/10.15468/dl.k5hxv2 |
| *Asplenium stuhlmannii* | GBIF.org (18 February 2024) GBIF Occurrence Download https://doi.org/10.15468/dl.2bncdt |
| *Cheilanthes inaequalis* | GBIF.org (22 March 2023) GBIF Occurrence Download https://doi.org/10.15468/dl.xba4pw |
| *Coleochloa abyssinica* | GBIF.org (28 May 2025) GBIF Occurrence Download https://doi.org/10.15468/dl.j3xsse |
| *Coleochloa domensis* | GBIF.org (28 May 2025) GBIF Occurrence Download https://doi.org/10.15468/dl.32hthf |
| *Cosentinia vellea* | GBIF.org (22 March 2023) GBIF Occurrence Download https://doi.org/10.15468/dl.dafh4z |
| *Craterostigma plantagineum* | GBIF.org (22 March 2023) GBIF Occurrence Download https://doi.org/10.15468/dl.pnxv3a |
| *Craterostigma yaundense* | GBIF.org (18 February 2024) GBIF Occurrence Download https://doi.org/10.15468/dl.huy7m8 |
| *Crepidomanes chevalieri* | GBIF.org (22 March 2023) GBIF Occurrence Download |

| | |
|---|---|
| | https://doi.org/10.15468/dl.we3am5 |
| *Crepidomanes melanotrichum* | GBIF.org (22 March 2023) GBIF Occurrence Download https://doi.org/10.15468/dl.r2sdq5 |
| *Didymoglossum erosum* | GBIF.org (22 March 2023) GBIF Occurrence Download https://doi.org/10.15468/dl.9avcdb |
| *Elaphoglossum acrostichoides* | GBIF.org (22 March 2023) GBIF Occurrence Download https://doi.org/10.15468/dl.698qkw |
| *Heminiotis farinosa* | GBIF.org (22 March 2023) GBIF Occurrence Download https://doi.org/10.15468/dl.vzzhtk |
| *Hymenophyllum capillare* | GBIF.org (22 March 2023) GBIF Occurrence Download https://doi.org/10.15468/dl.wqpewp |
| *Hymenophyllum hirsutum* | GBIF.org (22 March 2023) GBIF Occurrence Download https://doi.org/10.15468/dl.rr3cxv |
| *Hymenophyllum kuhnii* | GBIF.org (22 March 2023) GBIF Occurrence Download https://doi.org/10.15468/dl.f529nm |
| *Hymenophyllum splendidum* | GBIF.org (22 March 2023) GBIF Occurrence Download https://doi.org/10.15468/dl.7j4fhq |
| *Loxogramme abyssinica* | GBIF.org (22 March 2023) GBIF Occurrence Download https://doi.org/10.15468/dl.tdve7b |
| *Melpomene flabelliformis* | GBIF.org (22 March 2023) GBIF Occurrence Download https://doi.org/10.15468/dl.82wkxt |
| *Microchloa indica* | GBIF.org (22 March 2023) GBIF Occurrence Download https://doi.org/10.15468/dl.4pek7h |
| *Microchloa kunthii* | GBIF.org (22 March 2023) GBIF Occurrence Download https://doi.org/10.15468/dl.sym7kq |
| *Microdracoides squamosa* | GBIF.org (22 March 2023) GBIF Occurrence Download https://doi.org/10.15468/dl.6pwvgd |
| *Oropetium aristatum* | GBIF.org (22 March 2023) GBIF Occurrence Download https://doi.org/10.15468/dl.uuvqb7 |
| *Oropetium capense* | GBIF.org (22 March 2023) GBIF Occurrence Download https://doi.org/10.15468/dl.r6247a |
| *Pellaea dura* | GBIF.org (22 March 2023) GBIF Occurrence Download https://doi.org/10.15468/dl.zsgpw2 |
| *Phymatosorus scolopendria* | GBIF.org (18 February 2024) GBIF Occurrence Download https://doi.org/10.15468/dl.vk237u |
| *Platycerium stemaria* | GBIF.org (22 March 2023) GBIF Occurrence Download https://doi.org/10.15468/dl.ffzdam |
| *Pleopeltis macrocarpa* | GBIF.org (22 March 2023) GBIF Occurrence Download https://doi.org/10.15468/dl.ekk9fu |
| *Polyphlebium borbonicum* | GBIF.org (22 March 2023) GBIF Occurrence Download https://doi.org/10.15468/dl.zvdfg9 |
| *Selaginella njamnjamensis* | GBIF.org (22 March 2023) GBIF Occurrence Download https://doi.org/10.15468/dl.5uud4g |
| *Sporobolus festivus* | GBIF.org (18 February 2024) GBIF Occurrence Download https://doi.org/10.15468/dl.m8h3dp |
| *Sporobolus pellucidus* | GBIF.org (22 March 2023) GBIF Occurrence Download https://doi.org/10.15468/dl.7khsy2 |
| *Sporobolus stapfianus* | GBIF.org (22 March 2023) GBIF Occurrence Download https://doi.org/10.15468/dl.nx4pse |
| *Tripogon major* | GBIF.org (22 March 2023) GBIF Occurrence Download https://doi.org/10.15468/dl.pmnuqm |
| *Tripogon multiflorus* | GBIF.org (28 May 2025) GBIF Occurrence Download https://doi.org/10.15468/dl.9m6dnc |

| | |
|---|---|
| *Tripogonella minima* | GBIF.org (22 March 2023) GBIF Occurrence Download https://doi.org/10.15468/dl.gzfvm3 |
| *Vittaria guineensis* | GBIF.org (22 March 2023) GBIF Occurrence Download https://doi.org/10.15468/dl.8ng3dd |
| *Xerophyta schnizleinia* | GBIF.org (22 March 2023) GBIF Occurrence Download https://doi.org/10.15468/dl.suyyuq |

**Table S2.** Bioclimatic variables retrieved from CHELSA database and used in this study. 6

| Bioclimatic variables |
|---|
| BIO1 – Annual Mean Temperature |
| BIO2 – Mean Diurnal Range (Mean of monthly (max temp - min temp)) |
| BIO3 – Isothermality (BIO2/BIO7) (×100) |
| BIO4 – Temperature Seasonality (standard deviation ×100) |
| BIO5 – Max Temperature of Warmest Month |
| BIO7 – Temperature Annual Range (BIO5-BIO6) |
| BIO8 – Mean Temperature of Wettest Quarter |
| BIO12 – Annual Precipitation |
| BIO13 – Precipitation of Wettest Month |
| BIO14 – Precipitation of Driest Month |
| BIO15 – Precipitation Seasonality (Coefficient of Variation) |
| BIO16 – Precipitation of Wettest Quarter |
| BIO17 – Precipitation of Driest Quarter |
| BIO18 – Precipitation of Warmest Quarter |
| BIO19 – Precipitation of Coldest Quarter |

**Table S3.** List of R packages used in this study. 7

| Packages | References |
|---|---|
| dplyr | Wickham H, François R, Henry L, Müller K, Vaughan D (2023). dplyr: A Grammar of Data Manipulation. R package version 1.1.4, https://CRAN.R-project.org/package=dplyr |
| forcats | Wickham H (2023). forcats: Tools for Working with Categorical Variables (Factors).R package version 1.0.0, https://CRAN.R-project.org/package=forcats |
| ggplot2 | Wickham H (2016). ggplot2: Elegant Graphics for Data Analysis. Springer-Verlag New York. R package version 3.5.0, https://CRAN.R-project.org/package=ggplot2 |
| ggpubr | Kassambara A (2023). ggpubr: 'ggplot2' Based Publication Ready Plots. R package version 0.6.0, https://CRAN.R-project.org/package=ggpubr |
| mapdata | Brownrigg R, Minka TP, Deckmyn A (2023). mapdata: Extra Map Databases. R package version 2.3.1, https://CRAN.R-project.org/package=mapdata |
| maps | Becker RA, Wilks AR, Brownrigg R, Minka TP, Deckmyn A (2023). maps: Draw Geographical Maps. R package version 3.4.1, https://CRAN.R-project.org/package=maps |
| phytools | Revell LJ (2012). phytools: An R package for phylogenetic comparative biology (and other things). Methods in Ecology and Evolution, 3(2), 217–223. https://CRAN.R-project.org/package=phytools |
| rnaturalearth | South A (2017). rnaturalearth: World Map Data from Natural Earth. R package version 0.1.0, https://CRAN.R-project.org/package=rnaturalearth |
| sf | Pebesma E (2018). Simple Features for R: Standardized Support for Spatial Vector Data. R Journal, 10(1), 439–446. https://CRAN.R-project.org/package=sf |

| | |
|---|---|
| terra | Hijmans R (2023). terra: Spatial Data Analysis. R package version 1.7-65, <https://CRAN.R-project.org/package=terra> |
| V.PhyloMaker | Jin, Y & Qian, H. 2019. V.PhyloMaker: an R package that can generate very large phylogenies for vascular plants. Ecography. 42(8):1353–1359. doi.org/10.1111/ecog.04434 |
| viridis | Garnier S (2021). viridis: Default Color Maps from 'matplotlib'. R package version 0.6.4, https://CRAN.R-project.org/package=viridis |
| waffle | Rudis B, Gandy D 2023. waffle: Create Waffle Chart Visualizations. R package version 1.0.2, https://CRAN.R-project.org/package=waffle |

**Table S4.** Studies about desiccation-tolerant vascular plants in West Africa. Q1 - Did the study discuss conservation needs of DT plants?; Q2 - Did the study provide enough evidence that justifies the conservation needs of DT plants?; Q3 - Did the study propose effective conservation strategies for DT plants?; Categories: I - Studies focusing on the entire community; II - studies focusing on specific community components; III - studies focusing on ecological processes and functioning.

| References | Q1 | Q2 | Q3 | Categories |
|---|---|---|---|---|
| Gaff, D. F. (1986). Desiccation tolerant 'resurrection'grasses from Kenya and West Africa. Oecologia, 70, 118-120. | No | No | No | II |
| Krieger, A., Porembski, S., & Barthlott, W. (2000). Vegetation of seasonal rock pools on inselbergs situated in the savanna zone of the Ivory Coast (West Africa). Flora, 195(3), 257-266. | No | No | No | II |
| Müller, J. V. (2007). Herbaceous vegetation of seasonally wet habitats on inselbergs and lateritic crusts in West and Central Africa. Folia Geobotanica, 42, 29-61. | No | No | No | II |
| Oumorou, M., & Lejoly, J. (2003). Écologie, flore et végétation de l'inselberg Sobakperou (Nord-Bénin). Acta botanica gallica, 150(1), 65-84. | No | No | No | I |
| Owoseye, J. A., & Sanford, W. W. (1972). An ecological study of Vellozia schnitzleinia, a drought-enduring plant of northern Nigeria. The Journal of Ecology, 807-817. | No | No | No | II |
| Parmentier, I. (2001). Premières études sur la diversité végétale des inselbergs de Guinée Équatoriale continentale. Systematics and Geography of Plants, 911-922. | No | No | No | I |
| Parmentier, I., & Hardy, O. J. (2009). The impact of ecological differentiation and dispersal limitation on species turnover and phylogenetic structure of inselberg's plant communities. Ecography, 32(4), 613-622. | No | No | No | I |
| Parmentier, I., Oumorou, M., Pauwels, L., & Lejoly, J. (2006). Comparison of the ecology and distribution of the Poaceae flora on inselbergs embedded in savannah (Benin) or in rain forest (Western Central Africa). Belgian Journal of Botany, 65-77. | No | No | No | II |
| Porembski, S. (2000). The invasibility of tropical granite outcrops ('inselbergs') by exotic weeds. Journal of the Royal society of Western Australia, 83, 131. | Yes | No | No | III |
| Porembski, S. (2007). Tropical inselbergs: habitat types, adaptive strategies and diversity patterns. Brazilian Journal of Botany, 30, 579-586. | Yes | No | No | I |
| Porembski, S., & Barthlott, W. (1996). Plant species diversity of West African inselbergs. In The Biodiversity of African Plants: Proceedings XIVth AETFAT Congress 22–27 August 1994, Wageningen, The Netherlands (pp. 180-187). Springer | No | No | No | I |

| | | | | |
|---|---|---|---|---|
| Netherlands. | | | | |
| Porembski, S., & Barthlott, W. (1997). Seasonal Dynamics of Plant Diversity on lnselbergs in the Ivory Coast (West Africa). Botanica Acta, 110(6), 466-472. | No | No | No | I |
| Porembski, S., & Watve, A. (2005). Remarks on the species composition of ephemeral flush communities on paleotropical rock outcrops. Phytocoenologia, 389-402. | No | No | No | II |
| Porembski, S., Barthlott, W., Dörrstock, S., & Biedinger, N. (1994). Vegetation of rock outcrops in Guinea: granite inselbergs, sandstone table mountains and ferricretes—remarks on species numbers and endemism. Flora, 189(4), 315-326. | Yes | No | No | I |
| Porembski, S., Becker, U., & Seine, R. (2000). Islands on islands: habitats on inselbergs. In Inselbergs: biotic diversity of isolated rock outcrops in tropical and temperate regions (pp. 49-67). Berlin, Heidelberg: Springer Berlin Heidelberg. | No | No | No | II |
| Porembski, S., Brown, G., & Barthlott, W. (1996). A species-poor tropical sedge community: Afrotrilepis pilosa mats on inselbergs in West Africa. Nordic Journal of Botany, 16(3), 239-245. | No | No | No | II |
| Porembski, S., Seine, R., & Barthlott, W. (1997). Inselberg vegetation and the biodiversity of granite outcrops. Journal of the Royal Society of Western Australia, 80, 193. | No | No | No | I |
| Porembski, S., Silveira, F. A., Fiedler, P. L., Watve, A., Rabarimanarivo, M., Kouame, F., & Hopper, S. D. (2016). Worldwide destruction of inselbergs and related rock outcrops threatens a unique ecosystem. Biodiversity and Conservation, 25, 2827-2830. | Yes | No | No | III |
| Porembski, S., Szarzynski, J., Mund, J. P., & Barthlott, W. (1996). Biodiversity and vegetation of small-sized inselbergs in a West African rain forest (Taı, Ivory Coast). Journal of Biogeography, 23(1), 47-55. | No | No | No | I |
| Richards, P. W. (1957). Ecological notes on West African vegetation: I. The plant communities of the Idanre hills, Nigeria. The Journal of Ecology, 563-577. | No | No | No | I |
| Seine, R., Porembski, S., & Barthlott, W. (1996). A neglected habitat of carnivorous plants: inselbergs. Feddes Repertorium, 106(5-8), 555-562. | No | No | No | II |
| Szarzynski, J. (2000). Xeric islands: environmental conditions on inselbergs. In Inselbergs: biotic diversity of isolated rock outcrops in tropical and temperate regions (pp. 37-48). Berlin, Heidelberg: Springer Berlin Heidelberg. | No | No | No | I |
| Tindano, E., Ganaba, S., Sambare, O., & Thiombiano, A. (2015). Sahelian inselberg vegetation in Burkina Faso. *Bois & Forêts des Tropiques, 325*(3), 21–33. | No | No | No | I |
| Tindano, E., Kaboré, G. E., Porembski, S., & Thiombiano, A. (2024). Plant communities on inselbergs in Burkina Faso. Heliyon, 10(1). | No | No | No | I |
| Tindano, E., Kadéba, A., Traoré, I. C. E., & Thiombiano, A. (2023). Effects of abiotic factors on the flora and vegetation of inselbergs in Burkina Faso. Environmental Advances, 12, 100378. | No | No | No | I |
| Tindano, E., Lankoandé, B., Porembski, S., & Thiombiano, A. | No | No | No | I |

| | | | | | |
|---|---|---|---|---|---|
| (2023). Inselbergs: potential conservation areas for plant diversity in the face of anthropization. J. Phytol, 15, 70-79. | | | | | |
| Tindano, E., Poremski, S., Koehler, J., & Thiombiano, A. (2021). Ecological and floristic characterization of inselberg habitats in Burkina Faso. Geo-Eco-Trop, 45(4), 573-588. | No | No | No | | I |

**Table S5.** Species presence (1) and absence (0) across 123 inselbergs in West Africa. Ap – *Afrotrilepis pilosa*, As - *Asplenium stuhlmannii*, Cy - *Craterostigma yaundense*, Mi - *Microchloa indica*, Ms - *Microdracoides squamosa*, Oa - *Oropetium aristatum*, Pd - *Pellaea doniana*, Ps - *Phymathosorus scolopendria*, Sf - *Sporobolus festivus*, Tm - *Tripogonella minimus*.

| Inselbergs | Longitude | Latitude | Ap | As | Cy | Mi | Ms | Oa | Pd | Ps | Sf | Tm |
|---|---|---|---|---|---|---|---|---|---|---|---|---|
| Mt. Niangbo | -5.1775 | 8.8275 | 1 | 1 | 0 | 1 | 0 | 0 | 1 | 0 | 1 | 1 |
| Mt. Korhogo | -5.650833 | 9.4525 | 1 | 1 | 0 | 1 | 0 | 0 | 1 | 0 | 1 | 1 |
| Nambelegue | -5.675833 | 9.473889 | 1 | 1 | 0 | 1 | 0 | 0 | 1 | 0 | 1 | 1 |
| Near Korhogo | -5.615556 | 9.501111 | 1 | 1 | 0 | 1 | 0 | 0 | 1 | 0 | 1 | 1 |
| Boundiali 1 | -6.494444 | 9.534167 | 1 | 1 | 0 | 1 | 0 | 0 | 1 | 0 | 1 | 1 |
| Boundiali 2 | -6.626111 | 9.596667 | 1 | 1 | 0 | 1 | 0 | 0 | 1 | 0 | 1 | 1 |
| Boundiali 3 | -6.488056 | 9.493889 | 1 | 1 | 0 | 1 | 0 | 0 | 1 | 0 | 1 | 1 |
| Séguéla 1 | -6.544722 | 7.900278 | 1 | 1 | 0 | 1 | 0 | 0 | 1 | 0 | 1 | 1 |
| Séguéla 2 | -6.533333 | 7.9075 | 1 | 1 | 0 | 1 | 0 | 0 | 1 | 0 | 1 | 1 |
| Séguéla (road to Mankono) 1 | -6.638056 | 8.037222 | 1 | 1 | 0 | 1 | 0 | 0 | 1 | 0 | 1 | 1 |
| Séguéla (road to Mankono) 2 | -6.640556 | 8.029444 | 1 | 1 | 0 | 1 | 0 | 0 | 1 | 0 | 1 | 0 |
| Man (Cascade) | -7.628056 | 7.494722 | 1 | 1 | 0 | 0 | 0 | 0 | 1 | 1 | 1 | 0 |
| Danané | -8.131667 | 7.269444 | 1 | 1 | 0 | 0 | 0 | 0 | 1 | 1 | 1 | 0 |
| Duékoué 1 | -7.365556 | 6.756389 | 1 | 1 | 0 | 1 | 0 | 0 | 1 | 1 | 1 | 0 |
| Duékoué 2 | -7.376111 | 6.751111 | 1 | 1 | 0 | 1 | 0 | 0 | 1 | 1 | 1 | 0 |
| Man (Dent de Man) | -7.541667 | 7.452222 | 1 | 1 | 0 | 1 | 0 | 0 | 1 | 1 | 1 | 0 |
| Mt. Niénokoué | -7.17 | 5.434444 | 0 | 0 | 0 | 0 | 0 | 0 | 0 | 1 | 0 | 0 |
| Rocher d’Issia | -6.581111 | 6.483333 | 1 | 1 | 0 | 0 | 0 | 0 | 1 | 1 | 0 | 0 |
| Dabakala (Kadjeoule-Sourdi) | -4.546667 | 8.419444 | 1 | 1 | 0 | 1 | 0 | 0 | 1 | 0 | 1 | 1 |
| Nassian (Gbonkonou) | -3.500278 | 8.45 | 1 | 1 | 0 | 1 | 0 | 0 | 1 | 0 | 1 | 1 |
| Man (Cissus) | -7.575833 | 7.424167 | 1 | 1 | 0 | 1 | 0 | 0 | 1 | 1 | 1 | 0 |
| Tai-National Park | -7.209722 | 5.4625 | 0 | 0 | 0 | 0 | 0 | 0 | 0 | 1 | 0 | 0 |
| Duékoué (quarry) | -7.354167 | 6.754444 | 1 | 1 | 0 | 1 | 0 | 0 | 1 | 1 | 1 | 0 |
| Sénéma (south of Séguéla) | -6.58 | 7.7175 | 1 | 1 | 0 | 1 | 0 | 0 | 1 | 0 | 1 | 1 |
| near Mankono | -6.271667 | 8.098333 | 1 | 1 | 0 | 1 | 0 | 0 | 1 | 0 | 1 | 1 |
| near Bouaké | -5.112778 | 7.762222 | 1 | 1 | 0 | 1 | 0 | 0 | 1 | 0 | 1 | 1 |
| north of Boundiali | -6.466389 | 9.7275 | 1 | 1 | 0 | 1 | 0 | 0 | 1 | 0 | 1 | 1 |
| region of Abengourou (near Aniassué) | -3.717778 | 6.655 | 1 | 1 | 0 | 1 | 0 | 0 | 1 | 0 | 1 | 0 |
| region of Abengourou (near Atakro) | -3.810556 | 6.667778 | 1 | 1 | 0 | 1 | 0 | 0 | 1 | 0 | 1 | 0 |
| Mt. Mafa | -4.043611 | 5.8525 | 1 | 1 | 0 | 0 | 0 | 0 | 1 | 1 | 0 | 0 |
| near Foumbolo | -4.670833 | 8.586111 | 1 | 1 | 0 | 1 | 0 | 0 | 1 | 0 | 1 | 1 |
| Mt. Tonkoui | -7.643889 | 7.4425 | 1 | 1 | 0 | 0 | 0 | 0 | 1 | 1 | 1 | 0 |
| Comoé National Park, P 1 | -3.775 | 8.772222 | 1 | 1 | 0 | 1 | 0 | 0 | 1 | 0 | 1 | 1 |
| P 13, south of Kakpin | -3.777222 | 8.611389 | 1 | 1 | 0 | 1 | 0 | 0 | 1 | 0 | 1 | 1 |
| near Lolobo | -5.306667 | 6.969444 | 1 | 1 | 0 | 1 | 0 | 0 | 1 | 0 | 0 | 0 |
| west of Nassian | -3.4875 | 8.453611 | 1 | 1 | 0 | 1 | 0 | 0 | 1 | 0 | 1 | 1 |
| near Brobo | -4.828611 | 7.662222 | 1 | 1 | 0 | 1 | 0 | 0 | 1 | 0 | 0 | 1 |
| near Bouna (ferricrete) | -3.038611 | 9.354167 | 1 | 1 | 0 | 1 | 0 | 0 | 1 | 0 | 0 | 1 |
| near Odienné | -7.625556 | 9.679167 | 1 | 1 | 0 | 1 | 0 | 0 | 1 | 0 | 0 | 1 |
| near Tiémé 1 | -7.281667 | 9.554444 | 1 | 1 | 0 | 1 | 0 | 0 | 1 | 0 | 0 | 1 |
| near Tiémé 2 | -7.253889 | 9.559444 | 1 | 1 | 0 | 1 | 0 | 0 | 1 | 0 | 0 | 1 |

| | | | | | | | | | | | |
|---|---|---|---|---|---|---|---|---|---|---|---|
| near Badandougou | -7.156944 | 9.570278 | 1 | 1 | 0 | 1 | 0 | 0 | 1 | 0 | 0 | 1 |
| near Madinani 1 | -7.013333 | 9.629167 | 1 | 1 | 0 | 1 | 0 | 0 | 1 | 0 | 0 | 1 |
| near Madinani 2 | -6.813333 | 9.594444 | 1 | 1 | 0 | 1 | 0 | 0 | 1 | 0 | 0 | 1 |
| near Madinani 3 | -6.737778 | 9.595556 | 1 | 1 | 0 | 1 | 0 | 0 | 1 | 0 | 0 | 1 |
| near Gbando 1 | -6.668333 | 9.556389 | 1 | 1 | 0 | 1 | 0 | 0 | 1 | 0 | 0 | 1 |
| near Gbando 2 | -6.659722 | 9.544167 | 1 | 1 | 0 | 1 | 0 | 0 | 1 | 0 | 0 | 1 |
| near Touba (sandstone outcrop) | -7.631111 | 8.228056 | 1 | 1 | 0 | 1 | 0 | 0 | 1 | 0 | 1 | 1 |
| Daloa 1 | -6.434722 | 6.829444 | 1 | 1 | 0 | 0 | 0 | 0 | 1 | 0 | 0 | 0 |
| Daloa 2 | -6.434167 | 6.849722 | 1 | 1 | 0 | 0 | 0 | 0 | 1 | 0 | 1 | 0 |
| Sikensi 1 | -4.561667 | 5.645 | 1 | 1 | 0 | 0 | 0 | 0 | 1 | 1 | 0 | 0 |
| Sikensi 3 | -4.5575 | 5.653611 | 1 | 1 | 0 | 0 | 0 | 0 | 1 | 1 | 0 | 0 |
| near Tehini | -3.608056 | 9.595278 | 1 | 1 | 0 | 1 | 0 | 0 | 1 | 0 | 1 | 1 |
| near Tehini (ferricrete) | -3.58 | 9.607778 | 1 | 1 | 0 | 1 | 0 | 0 | 1 | 0 | 1 | 1 |
| near Tondoura (BF) | -4.773611 | 10.173889 | 0 | 0 | 0 | 1 | 0 | 0 | 1 | 0 | 1 | 1 |
| near Mangodara (BF) | -4.441667 | 9.857222 | 1 | 1 | 0 | 1 | 0 | 0 | 1 | 0 | 1 | 1 |
| near Wayen (BF) | -0.983333 | 12.323611 | 0 | 0 | 0 | 1 | 0 | 1 | 0 | 0 | 1 | 1 |
| near Zorgho (BF) | -0.632222 | 12.211111 | 0 | 0 | 0 | 1 | 0 | 1 | 0 | 0 | 1 | 1 |
| near Léo (BF) | -2.193611 | 11.115833 | 0 | 0 | 0 | 1 | 0 | 1 | 1 | 0 | 1 | 1 |
| Nazinga (BF) | -1.6075 | 11.178611 | 0 | 0 | 0 | 1 | 0 | 1 | 1 | 0 | 1 | 1 |
| near Bobo-Dioulasso | -4.135278 | 11.303611 | 0 | 0 | 0 | 1 | 0 | 1 | 1 | 0 | 1 | 1 |
| near Banfora (BF) sandstone | -4.595833 | 10.848889 | 1 | 1 | 0 | 1 | 0 | 0 | 1 | 0 | 1 | 1 |
| Reserve de Bontioli (BF) ferricrete | -2.955556 | 10.944167 | 0 | 0 | 0 | 1 | 0 | 1 | 0 | 0 | 1 | 1 |
| near Po 1 | -1.123056 | 11.124722 | 0 | 0 | 0 | 1 | 0 | 1 | 1 | 0 | 1 | 1 |
| near Po 2 | -1.084167 | 11.073611 | 0 | 0 | 0 | 1 | 0 | 1 | 0 | 0 | 1 | 1 |
| Soubakpérou (Benin) | 2.160278 | 9.144444 | 1 | 1 | 0 | 1 | 0 | 0 | 1 | 0 | 1 | 1 |
| near Savé (Benin) | 2.163056 | 9.144444 | 1 | 1 | 0 | 1 | 0 | 0 | 1 | 0 | 1 | 1 |
| near Kandi 1 | 2.895556 | 11.130833 | 0 | 0 | 0 | 1 | 0 | 1 | 0 | 0 | 1 | 1 |
| near Kandi 2 | 2.900278 | 11.108333 | 0 | 0 | 0 | 1 | 0 | 1 | 0 | 0 | 1 | 1 |
| near Goungoun (ferricrete) 1 | 3.154444 | 11.574167 | 0 | 0 | 0 | 1 | 0 | 1 | 0 | 0 | 1 | 1 |
| near Goungoun (ferricrete) 2 | 3.150556 | 11.565 | 0 | 0 | 0 | 1 | 0 | 1 | 0 | 0 | 1 | 1 |
| near Goungoun (ferricrete) 3 | 3.160278 | 11.543333 | 0 | 0 | 0 | 1 | 0 | 1 | 1 | 0 | 1 | 1 |
| Dassa 1 | 2.190833 | 7.782222 | 1 | 1 | 0 | 1 | 0 | 0 | 1 | 0 | 1 | 1 |
| Dassa 2 | 2.195278 | 7.753333 | 1 | 1 | 0 | 1 | 0 | 0 | 1 | 0 | 1 | 1 |
| Dassa 3 | 2.202222 | 7.709722 | 1 | 1 | 0 | 1 | 0 | 0 | 1 | 0 | 1 | 1 |
| Savalou | 1.978333 | 7.965833 | 1 | 1 | 0 | 1 | 0 | 0 | 1 | 0 | 1 | 1 |
| Natitingou (sandstone) 1 | 1.369167 | 10.306389 | 1 | 1 | 0 | 1 | 0 | 0 | 1 | 0 | 1 | 1 |
| Natitingou (sandstone) 2 | 1.395 | 10.301111 | 1 | 1 | 0 | 1 | 0 | 0 | 1 | 0 | 1 | 1 |
| near Natitingou (sandstone) | 1.444167 | 10.210833 | 1 | 1 | 0 | 1 | 0 | 0 | 1 | 0 | 1 | 1 |
| near Biguina | 1.696667 | 8.764722 | 1 | 1 | 0 | 1 | 0 | 0 | 1 | 0 | 1 | 1 |
| Savè | 2.508056 | 8.036389 | 1 | 1 | 0 | 1 | 0 | 0 | 1 | 0 | 1 | 1 |
| near Savè 1 | 2.581944 | 8.04 | 1 | 1 | 0 | 1 | 0 | 0 | 1 | 0 | 1 | 1 |
| near Savè 2 | 2.61 | 8.044722 | 1 | 1 | 0 | 1 | 0 | 0 | 1 | 0 | 1 | 1 |
| near Gorobani: | 2.026389 | 9.4775 | 1 | 1 | 0 | 1 | 0 | 0 | 1 | 0 | 1 | 1 |
| near Yebessi | 2.105278 | 9.346667 | 1 | 1 | 0 | 1 | 0 | 0 | 1 | 0 | 1 | 1 |
| Yebessi | 2.133056 | 9.326111 | 1 | 1 | 0 | 1 | 0 | 0 | 1 | 0 | 1 | 1 |
| near Kpéssou | 2.196111 | 9.296111 | 1 | 1 | 0 | 1 | 0 | 0 | 1 | 0 | 1 | 1 |
| Kpéssou | 2.188611 | 9.285556 | 1 | 1 | 0 | 1 | 0 | 0 | 1 | 0 | 1 | 1 |
| Yaounde 1 | 11.393333 | 3.828056 | 0 | 0 | 1 | 0 | 1 | 0 | 1 | 1 | 0 | 0 |
| Yaounde 2 | 11.425556 | 3.830278 | 0 | 0 | 1 | 0 | 1 | 0 | 1 | 1 | 0 | 0 |
| Yaounde 3 | 11.434167 | 3.887778 | 0 | 0 | 1 | 0 | 1 | 0 | 1 | 1 | 0 | 0 |
| Yaounde 4 | 11.443056 | 3.827222 | 0 | 0 | 1 | 0 | 1 | 0 | 1 | 1 | 0 | 0 |
| Yaounde 5 | 11.466389 | 3.853056 | 0 | 0 | 1 | 0 | 1 | 0 | 1 | 1 | 0 | 0 |
| Mamfe 1 | 9.322778 | 5.766111 | 1 | 1 | 0 | 0 | 1 | 0 | 1 | 1 | 1 | 0 |

| | | | | | | | | | | | |
|---|---|---|---|---|---|---|---|---|---|---|---|
| Mamfe 2 | 9.336944 | 5.758333 | 1 | 1 | 0 | 0 | 1 | 0 | 1 | 1 | 1 | 0 |
| Takamanda 1 | 9.513889 | 6.175833 | 0 | 0 | 0 | 0 | 1 | 0 | 1 | 1 | 1 | 0 |
| Takamanda 2 | 9.525833 | 6.111944 | 0 | 0 | 0 | 0 | 1 | 0 | 1 | 1 | 1 | 0 |
| Friguiagabe (sandstone outcrop) | -12.912778 | 9.976389 | 1 | 1 | 0 | 1 | 1 | 0 | 1 | 0 | 1 | 0 |
| Mt. Gangan (sandstone outcrop) 1 | -12.893056 | 10.055 | 1 | 1 | 0 | 1 | 0 | 0 | 1 | 0 | 1 | 0 |
| Mt. Gangan (sandstone outcrop) 2 | -12.878611 | 10.078056 | 1 | 1 | 0 | 1 | 0 | 0 | 1 | 0 | 1 | 0 |
| Macenta | -9.460556 | 8.522222 | 1 | 1 | 0 | 0 | 0 | 0 | 1 | 0 | 1 | 0 |
| near Macenta | -9.491667 | 8.599167 | 1 | 1 | 0 | 0 | 0 | 0 | 1 | 0 | 1 | 0 |
| near Balizia | -9.605 | 8.601111 | 1 | 1 | 0 | 0 | 0 | 0 | 1 | 0 | 1 | 0 |
| Guéckédou | -10.098611 | 8.557222 | 1 | 1 | 0 | 1 | 0 | 0 | 1 | 1 | 1 | 0 |
| near Kolobingo | -10.017222 | 8.553611 | 1 | 1 | 0 | 0 | 0 | 0 | 1 | 1 | 1 | 0 |
| near Técoulo | -9.984444 | 8.534167 | 1 | 1 | 0 | 0 | 0 | 0 | 1 | 1 | 1 | 0 |
| Tongo Hills 1 | -0.812778 | 10.67 | 0 | 0 | 0 | 1 | 0 | 1 | 0 | 0 | 1 | 1 |
| Tongo Hills 2 | -0.803611 | 10.688889 | 0 | 0 | 0 | 1 | 0 | 1 | 0 | 0 | 1 | 1 |
| Akure | 5.181944 | 7.216389 | 1 | 1 | 0 | 1 | 0 | 0 | 1 | 1 | 1 | 0 |
| Near Akure 1 | 5.161389 | 7.343333 | 1 | 1 | 0 | 1 | 0 | 0 | 1 | 1 | 1 | 0 |
| Near Akure 2 | 5.171389 | 7.348611 | 1 | 1 | 0 | 1 | 0 | 0 | 1 | 1 | 1 | 0 |
| Near Akure 3 | 5.226389 | 7.374167 | 1 | 1 | 0 | 1 | 0 | 0 | 1 | 1 | 1 | 0 |
| Idanre 1 | 5.15 | 7.108889 | 1 | 1 | 0 | 0 | 0 | 0 | 1 | 1 | 1 | 0 |
| Idanre 2 | 5.144722 | 7.111389 | 1 | 1 | 0 | 0 | 0 | 0 | 1 | 1 | 1 | 0 |
| Idanre 3 | 5.133056 | 7.110833 | 1 | 1 | 0 | 0 | 0 | 0 | 1 | 1 | 1 | 0 |
| Idanre 4 | 5.105556 | 7.126667 | 1 | 1 | 0 | 1 | 0 | 0 | 1 | 1 | 1 | 0 |
| near Idanre 1 | 5.034167 | 7.175278 | 1 | 1 | 0 | 1 | 0 | 0 | 1 | 1 | 1 | 0 |
| near Idanre 2 | 5.0425 | 7.190278 | 1 | 1 | 0 | 1 | 0 | 0 | 1 | 1 | 1 | 0 |
| Bicurga | 10.471111 | 1.583611 | 0 | 0 | 0 | 0 | 0 | 0 | 1 | 1 | 0 | 0 |
| Piedras Nzas 1 | 11.031944 | 1.456389 | 0 | 0 | 0 | 0 | 0 | 0 | 1 | 1 | 1 | 0 |
| Piedras Nzas 2 | 11.021667 | 1.463889 | 0 | 0 | 0 | 0 | 0 | 0 | 1 | 1 | 1 | 0 |
| Dumu | 11.323611 | 1.368056 | 0 | 0 | 0 | 0 | 0 | 0 | 1 | 1 | 1 | 0 |
| near Asoc | 11.276944 | 1.451111 | 0 | 0 | 0 | 0 | 0 | 0 | 1 | 1 | 1 | 0 |

**Table S6.** Species exposure to two anthropogenic drivers of biodiversity loss: quarrying and climate change. The evaluated species are *Asplenium stuhlmannii* (Aspleniaceae), *Phymathosorus scolopendria* (Polypodiaceae), *Pellaea doniana* (Pteridaceae), *Afrotrilepis pilosa* and *Microdracoides squamosa* (Cyperaceae), *Microchloa indica, Oropetium aristatum, Tripogonella minimus*, and *Sporobolus festivus* (Poaceae), and *Craterostigma yaundense* (Linderniaceae).

| **Species** | **Exposure** | | |
|---|---|---|---|
| **Quarrying** | | | |
| *A. pilosa* | 10.75% (10 out of 93) | | |
| *A. stuhlmannii* | 10.75% (10 out of 93) | | |
| *C. yaundense* | 60% (3 out of 5) | | |
| *M. indica* | 8.89% (8 out of 90) | | |
| *M. squamosa* | 40% (4 out of 10) | | |
| *O. aristatum* | 13.33% (2 out of 15) | | |
| *P. doniana* | 13.64% (15 out of 110) | | |
| *P. scolopendria* | 14.63% (6 out of 41) | | |
| *S. festivus* | 8.26% (9 out of 109) | | |
| *T. minimus* | 7.04% (5 out of 71) | | |
| **Climate change** | **2011-2040** | **2041-2070** | **2071_2100** |
| **SSP1** | | | |
| *A. pilosa* | 0.5 (0.4 - 0.6) | 1.4 (1.2 - 1.6) | 1.4 (1.2 - 1.5) |
| *A. stuhlmannii* | 0.6 (0.5 - 0.8) | 1.7 (1.6 - 2) | 1.8 (1.6 - 2) |
| *C. yaundense* | 1.2 (1.2 - 1.2) | 3.5 (3.4 - 3.5) | 3.8 (3.7 - 3.9) |

| | | | |
|---|---|---|---|
| *M. indica* | 0.06 (0 - 0.1) | 0.1 (0.1 - 0.1) | 0.1 (0.1 - 0.2) |
| *M. squamosa* | 0.9 (0.8 - 0.9) | 2.2 (2.1 - 2.3) | 2.4 (2.2 - 2.5) |
| *O. aristatum* | 0.5 (0.4 - 0.6) | 0.6 (0.6 - 0.8) | 1.4 (1.3 - 1.6) |
| *P. doniana* | 0.2 (0.1 - 0.3) | 0.2 (0.2 - 0.3) | 0.5 (0.3 - 0.6) |
| *P. scolopendria* | 0.4 (0.3 - 0.5) | 1.1 (1 - 1.2) | 1.2 (1 - 1.4) |
| *S. festivus* | 0.1 (0.1 - 0.2) | 0.4 (0.3 - 0.4) | 0.4 (0.3 - 0.5) |
| *T. minimus* | 0.1 (0.1 - 0.2) | 0.4 (0.4 - 0.5) | 0.4 (0.3 - 0.5) |
| **SSP5** | | | |
| *A. pilosa* | 0.6 (0.5 - 0.7) | 3.9 (3.5 - 4.3) | 13.2 (11 - 14) |
| *A. stuhlmannii* | 0.8 (0.6 - 0.9) | 5 (4.3 - 5.4) | 16.6 (14 - 18) |
| *C. yaundense* | 1.4 (1.4 - 1.4) | 9.4 (9.3 - 9.4) | 28.2 (28 - 28) |
| *M. indica* | 0.06 (0 - 0.1) | 0.3 (0.3 - 0.4) | 1.1 (0.9 - 1.2) |
| *M. squamosa* | 1 (0.9 - 1.1) | 6.5 (6.1 - 6.7) | 21.4 (20 - 22) |
| *O. aristatum* | 0.7 (0.7 - 0.9) | 4.1 (3.9 - 4.8) | 14.9 (14 - 17) |
| *P. doniana* | 0.2 (0.1 - 0.3) | 1.3 (0.9 - 1.5) | 4.3 (3.7 - 4.8) |
| *P. scolopendria* | 0.5 (0.3 - 0.6) | 2.9 (2.5 - 3.2) | 9.1 (7.9 - 9.7) |
| *S. festivus* | 0.4 (0.3 - 0.5) | 1 (0.8 - 1.2) | 3.4 (3 - 4) |
| *T. minimus* | 0.2 (0.1 - 0.2) | 1.2 (0.9 - 1.3) | 3.9 (3.4 - 4.6) |

**Table S7.** Species exposure to quarrying when comparing unprotected and protected inselbergs. The evaluated species are *Asplenium stuhlmannii* (Aspleniaceae), *Phymathosorus scolopendria* (Polypodiaceae), *Pellaea doniana* (Pteridaceae), *Afrotrilepis pilosa* and *Microdracoides squamosa* (Cyperaceae), *Microchloa indica*, *Oropetium aristatum*, *Tripogonella minimus*, and *Sporobolus festivus* (Poaceae), and *Craterostigma yaundense* (Linderniaceae).

| Species | Number of unprotected inselbergs | | Number of protected inselbergs | |
|---|---|---|---|---|
| | **Without quarrying** | **With quarrying** | **Without quarrying** | **With quarrying** |
| *A. pilosa* | 69 (88%) | 9 (12%) | 14 (93%) | 1 (7%) |
| *A. stuhlmannii* | 69 (88%) | 9 (12%) | 14 (93%) | 1 (7%) |
| *C. yaundense* | 2 (40%) | 3 (60%) | - | - |
| *M. indica* | 65 (90%) | 7 (10%) | 17 (94%) | 1 (6%) |
| *M. squamosa* | 6 (60%) | 4 (40%) | - | - |
| *O. aristatum* | 9 (82%) | 2 (18%) | 4 (100%) | 0 (0%) |
| *P. doniana* | 78 (85%) | 14 (15%) | 17 (94%) | 1 (6%) |
| *P. scolopendria* | 30 (83%) | 6 (17%) | 5 (100%) | 0 (0%) |
| *S. festivus* | 80 (91%) | 8 (9%) | 20 (95%) | 1 (5%) |
| *T. minimus* | 51 (93%) | 4 (7%) | 15 (94%) | 1 (6%) |

**Table S8.** Species exposure to climate change when comparing unprotected and protected inselbergs. The evaluated species are *Asplenium stuhlmannii* (Aspleniaceae), *Phymathosorus scolopendria* (Polypodiaceae), *Pellaea doniana* (Pteridaceae), *Afrotrilepis pilosa* and *Microdracoides squamosa* (Cyperaceae), *Microchloa indica*, *Oropetium aristatum*, *Tripogonella minimus*, and *Sporobolus festivus* (Poaceae), and *Craterostigma yaundense* (Linderniaceae).

| Species | Unprotected inselbergs | | | Protected inselbergs | | |
|---|---|---|---|---|---|---|
| | **2011-2040** | **2041-2070** | **2071_2100** | **2011-2041** | **2041-2071** | **2071_2101** |
| **SSP1** | | | | | | |
| *A. pilosa* | 0.5 (0.4 - 0.6) | 1.4 (1.3 - 1.6) | 1.4 (1.2 - 1.5) | 0.5 (0.4 - 0.5) | 1.3 (1.2 - 1.4) | 1.3 (1.2 - 1.4) |
| *A. stuhlmannii* | 0.6 (0.5 - 0.8) | 1.7 (1.6 - 2) | 1.8 (1.6 - 2) | 0.6 (0.5 - 0.7) | 1.7 (1.6 - 1.8) | 1.7 (1.6 - 1.9) |
| *C. yaundense* | 1.2 (1.2 - 1.2) | 3.5 (3.4 - 3.5) | 3.9 (3.7 - 3.9) | - | - | - |
| *M. indica* | 0.06 (< 0.1) | 0.1 (0.1 - 0.1) | 0.1 (0.1 - 0.2) | 0.05 (< 0.1) | 0.1 (0.1 - 0.1) | 0.1 (0.1 - 0.1) |
| *M. squamosa* | 0.9 (0.8 - 0.9) | 2.2 (2.1 - 2.3) | 2.4 (2.2 - 2.5) | - | - | - |
| *O. aristatum* | 0.5 (0.4 - 0.6) | 0.6 (0.6 - 0.8) | 1.4 (1.3 - 1.6) | 0.4 (0.4 - 0.4) | 0.6 (0.6 - 0.6) | 1 (1 - 1.4) |

| | | | | | | |
|---|---|---|---|---|---|---|
| *P. doniana* | 0.2 (0.1 - 0.3) | 0.2 (0.2 - 0.3) | 0.5 (0.3 - 0.6) | 0.2 (0.1 - 0.2) | 0.2 (0.2 - 0.2) | 0.5 (0.5 - 0.5) |
| *P. scolopendria* | 0.4 (0.3 - 0.5) | 1.1 (1 - 1.2) | 1.2 (1 - 1.4) | 0.4 (0.4 - 0.4) | 1.1 (1.1 - 1.1) | 1.2 (1 - 1.2) |
| *S. festivus* | 0.1 (0.1 - 0.2) | 0.4 (0.3 - 0.4) | 0.4 (0.3 - 0.5) | 0.1 (0.1 - 0.1) | 0.4 (0.3 - 0.4) | 0.4 (0.4 - 0.4) |
| *T. minimus* | 0.2 (0.1 - 0.2) | 0.4 (0.4 - 0.5) | 0.4 (0.3 - 0.5) | 0.1 (0.1 - 0.2) | 0.4 (0.4 - 0.4) | 0.4 (0.4 - 0.4) |
| **SSP5** | | | | | | |
| *A. pilosa* | 0.6 (0.5 - 0.7) | 4 (3.5 - 4.3) | 13.2 (11 - 14) | 0.6 (0.5 - 0.7) | 3.9 (3.6 - 4.1) | 13.2 (12 - 13) |
| *A. stuhlmannii* | 0.8 (0.6 - 0.9) | 5 (4.3 - 5.4) | 16.6 (14 - 18) | 0.7 (0.6 - 0.8) | 4.9 (4.5 - 5.1) | 16.7 (16 - 17) |
| *C. yaundense* | 1.4 (1.4 - 1.4) | 9.4 (9.3 - 9.4) | 28.2 (28 - 28) | - | - | - |
| *M. indica* | 0.1 (0 - 0.1) | 0.3 (0.3 - 0.4) | 1.1 (0.9 - 1.2) | 0.06 (0 - 0.1) | 0.3 (0.3 - 0.4) | 1.1 (1 - 1.1) |
| *M. squamosa* | 1 (0.9 - 1.1) | 6.5 (6.1 - 6.7) | 21.4 (20 - 22) | - | - | - |
| *O. aristatum* | 0.7 (0.7 - 0.9) | 4.1 (3.9 - 4.8) | 15 (14 - 17) | 0.7 (0.7 - 0.7) | 4 (3.9 - 4) | 14.5 (14 - 14) |
| *P. doniana* | 0.2 (0.1 - 0.3) | 1.3 (0.9 - 1.5) | 4.3 (3.7 - 4.8) | 0.2 (0.2 - 0.2) | 1.3 (1.2 - 1.3) | 4.3 (4.1 - 4.5) |
| *P. scolopendria* | 0.5 (0.3 - 0.6) | 2.9 (2.5 - 3.2) | 9.1 (7.9 - 9.7) | 0.4 (0.4 - 0.5) | 2.7 (2.5 - 2.9) | 8.7 (8.3 - 9.1) |
| *S. festivus* | 0.4 (0.3 - 0.5) | 1 (0.8 - 1.2) | 3.5 (3 - 4) | 0.4 (0.4 - 0.5) | 1 (0.9 - 1.1) | 3.4 (3.3 - 3.6) |
| *T. minimus* | 0.2 (0.1 - 0.2) | 1.2 (0.9 - 1.3) | 4 (3.4 - 4.6) | 0.2 (0.1 - 0.2) | 1.1 (1 - 1.2) | 3.9 (3.7 - 4) |
